# Evaluation of deep and shallow learning methods in chemogenomics for the prediction of drugs specificity

**DOI:** 10.1186/s13321-020-0413-0

**Published:** 2020-02-10

**Authors:** Benoit Playe, Veronique Stoven

**Affiliations:** 1 Center for Computational Biology, Mines ParisTech, PSL Research University, 60 Bd Saint-Michel, 75006 Paris, France; 2 Institut Curie, 75248 Paris, France; 3 INSERM U900, 75248 Paris, France

**Keywords:** Chemogenomics, Drug virtual screening, Deep learning, Graph neural networks

## Abstract

Chemogenomics, also called proteochemometrics, covers a range of computational methods that can be used to predict protein–ligand interactions at large scales in the protein and chemical spaces. They differ from more classical ligand-based methods (also called QSAR) that predict ligands for a given protein receptor. In the context of drug discovery process, chemogenomics allows to tackle the question of predicting off-target proteins for drug candidates, one of the main causes of undesirable side-effects and failure within drugs development processes. The present study compares shallow and deep machine-learning approaches for chemogenomics, and explores data augmentation techniques for deep learning algorithms in chemogenomics. Shallow machine-learning algorithms rely on expert-based chemical and protein descriptors, while recent developments in deep learning algorithms enable to learn abstract numerical representations of molecular graphs and protein sequences, in order to optimise the performance of the prediction task. We first propose a formulation of chemogenomics with deep learning, called the chemogenomic neural network (CN), as a feed-forward neural network taking as input the combination of molecule and protein representations learnt by molecular graph and protein sequence encoders. We show that, on large datasets, the deep learning CN model outperforms state-of-the-art shallow methods, and competes with deep methods with expert-based descriptors. However, on small datasets, shallow methods present better prediction performance than deep learning methods. Then, we evaluate data augmentation techniques, namely multi-view and transfer learning, to improve the prediction performance of the chemogenomic neural network. We conclude that a promising research direction is to integrate heterogeneous sources of data such as auxiliary tasks for which large datasets are available, or independently, multiple molecule and protein attribute views.

## Introduction

### Interest of chemogenomics in drug discovery

The current paradigm in rationalised drug design usually associates a disease to one or several protein targets, called therapeutic targets, belonging to biological pathways that are involved in the disease development. The goal of the drug discovery process is then to identify a drug molecule that binds to the protein target and alters disease evolution.

However, the drug discovery process has limited success, and only a few tens of new drugs reach the market every year. Indeed, most of the hits identified by high-throughput screening (HTS) fail to become approved drugs because of unwanted side effects and toxicity. This in part results from unexpected interactions with so called “off-target” proteins due to drugs lack of specificity.

However, it is impossible to conduct bio-assays at the human proteome scale to discard molecules with unacceptable off-targets [1, 2], and chemoinformatics [3] algorithms provide interesting in silico screening methods. Chemogenomics is a generalisation of Quantitative Structure-Activity Relationship (QSAR) methods [4]. QSAR methods can predict interactions for a given protein (or few, in the case of multi-task QSAR), while chemogenomic models are trained to simultaneously predict interactions for several proteins [5], with the underlying idea that the prediction of a drug–target interaction may benefit from interactions known between other targets and other molecules. Chemogenomics enables to predict drugs unexpected “off-targets”, and guide experiments to only test interactions predicted with high probability scores. Note that some authors use the word “proteochemometrics” as a synonym for chemogenomics, and various studies report the diversity of applications of proteochemometrics [6]. The word “proteochemometrics” is often preferred in papers that study the impact of protein and molecule descriptors on prediction performances [7, 8]. In the present paper, we tackle the question of predicting drugs specificity at large scale in the space of proteins, and therefore, the word “chemogenomics” appeared more adequate. We explore chemogenomics with deep learning approaches, including transfer learning strategies, and compare their prediction performances to those of state-of-the-art shallow methods. We formulate the problem as a Drug–Target Interaction (DTI) prediction task, in terms of a binary classification of (protein,molecule) pairs that interact or not.

### Outline and contributions

“Reference shallow methods in machine-learning for chemogenomics” section recalls key reference shallow machine-learning methods for chemogenomics. In “Proposed chemogenomic neural network” section, we present a formal scheme, named the chemogenomic neural network (CN), for molecule and protein representation learning in the context deep learning for chemogenomics. “Related works” section shortly reviews related works in chemogenomics and QSAR with deep learning approaches. It is followed by the “Materials and methods” section.

Then, in the “Results” section, we first compare the prediction performances of state-of-the-art shallow and deep machine-learning methods, which was never discussed in previous chemogenomics studies. We show that, on small datasets, the more simple and less computationally demanding shallow methods perform better than deep learning methods. On large datasets, the proposed chemogenomic neural network (CN) with representation learning competes with state-of-the-art shallow and deep methods that use expert-based descriptors, but is not ultimately superior. These conclusions differ from those of previous works which considered specific datasets and compared deep learning approaches with baseline (i.e. not state-of-the-art) shallow methods.

Furthermore, we consider data augmentation techniques, namely multi-view and transfer learning. In particular, we tested the interest of combining expert-based and learnt features. We also tested various implementations of transfer learning using additional larger datasets to pre-train the encoders, before their use in the chemogenomic task of interest. We show that multi-view and transfer learning can improve prediction performance of deep learning methods on small datasets, although not reaching the performances of shallow methods. However, for large datasets, we also show that a less sophisticated model in which the deep neural network simply uses expert-based molecule and protein descriptors (i.e. the descriptors that are not learnt), outperforms state-of-the-art shallow models.

We finally present a “Discussion and conclusion” section about the proposed methods for chemogenomics with deep learning.

## Reference shallow methods in machine-learning for chemogenomics

Various chemogenomics methods have been proposed in the last decade [9–23]. They differ by (i) the descriptors used to encode proteins and ligands (ii) how similarities are measured between these objects, (iii) the machine-learning algorithm that is used to learn the model and make the predictions.

Jacob and Vert [5] used the Kronecker product of protein and ligand kernels to define the kernel associated with the chemogenomic space, i.e. the space of (protein,molecule) pairs. This approach has been successfully applied to DTI prediction [10, 24, 25]. In this paper, this method called “kronSVM” is used it as reference to benchmark the considered deep learning algorithms for chemogenomics.

As another reference shallow model, we also used Matrix factorisation (MF) approaches that decompose the (protein, molecule) interaction matrix into the product of two matrices of lower ranks that operate in the two corresponding latent spaces of proteins and molecules [22, 26]. More precisely, we used the *NRLMF* method developed by Liu et al [23], because it proved to outperform other shallow state-of-the-art methods on various datasets [9, 18–20, 22].

## Proposed chemogenomic neural network

### Overall scheme for the chemogenomic neural network

**Fig. 1:**
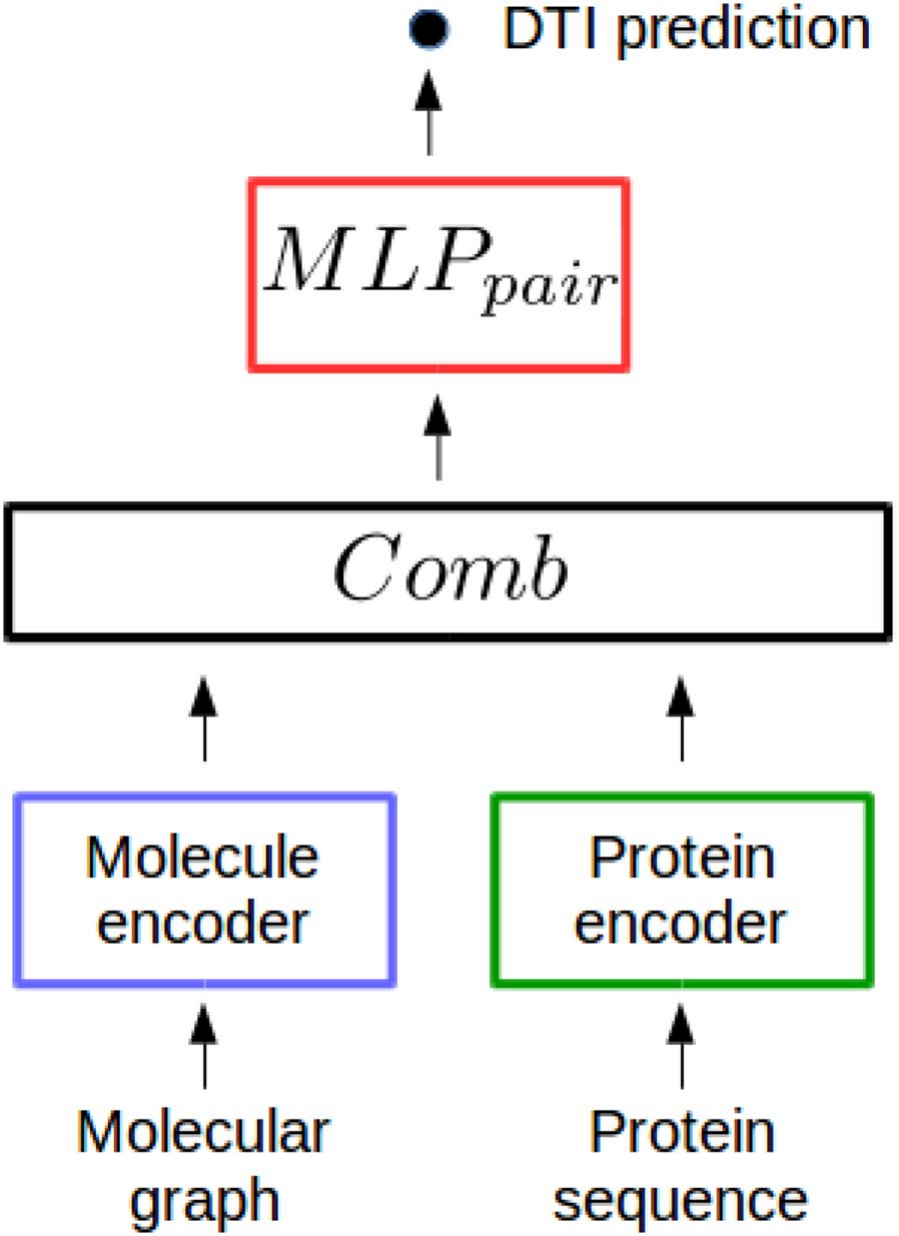
The chemogenomic neural network (CN)

Our general scheme for chemogenomics with deep learning is represented in Fig. 1. It contains four main building blocks : (1) a molecule encoder that learns abstract descriptors for molecules based on their structure (2) a protein encoder that learns abstract descriptors from their amino acids sequence, (3) a *Comb* block, i.e. an operation or a neural network module that combines molecule and protein descriptors to build a pairwise latent representation for the (molecule, protein) pair, and (4) the $MLP_{pair}$ (for Multi Layer Perceptron on pairs, also called feed-forward neural network, FNN) that predicts if the (molecule,protein) pairs interact.

Any protein sequence and molecular graph encoders can be considered, and the four building blocks are detailed in the next sections.

### Molecular graph encoder

Classically, molecules are represented by a one dimensional representation, such as the SMILES [27] representation, which can be encoded by recurrent neural networks.

Molecules can also be represented in 2D by their molecular graphs $G=(\mathcal {V},\mathcal {E})$, where $\mathcal {V}$ is the set of vertices (or nodes), and $\mathcal {E}$ the set of edges. Nodes are attributed with atom properties (like atom type, or physicochemical properties), and edges are attributed with bond properties (like bond type, or topological properties). Such undirected graphs can be encoded by Graph Neural Networks (GNN). Approaches based on recurrent neural networks processing SMILES proved to be competitive with GNN processing the molecular graph [28, 29]. However, in the present study, we used GNNs because they offer a wider implementation versatility and larger room for improvements.

Each node *i* and each edge (*i*, *j*) respectively has input attribute vectors $\mathbf {x}_i$ and $\mathbf {x}_{ij}$ (in our case, descriptors of atoms and bonds). We note $\mathbf {h}_i$ and $\mathbf {h}_{ij}$ the node and edge representations learnt by the molecule encoder. $\mathcal {N}(i)$ refers to the neighbouring nodes of node *i*, which can be the one-hop neighbourhood (i.e. the nodes reached by paths of length 1, which we used in practice), and *L* represents the total number of neural layers in the GNN.

Alg. 1 presents the general algorithm we used for GNN, which is inspired from Hamilton et al. [30, 31].

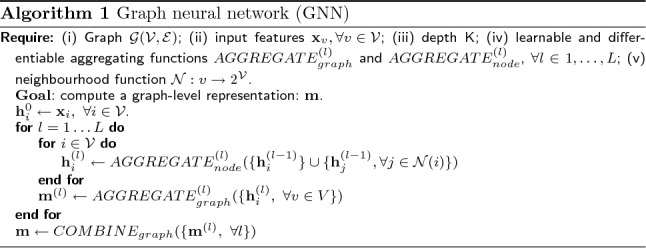



**Fig. 2:**
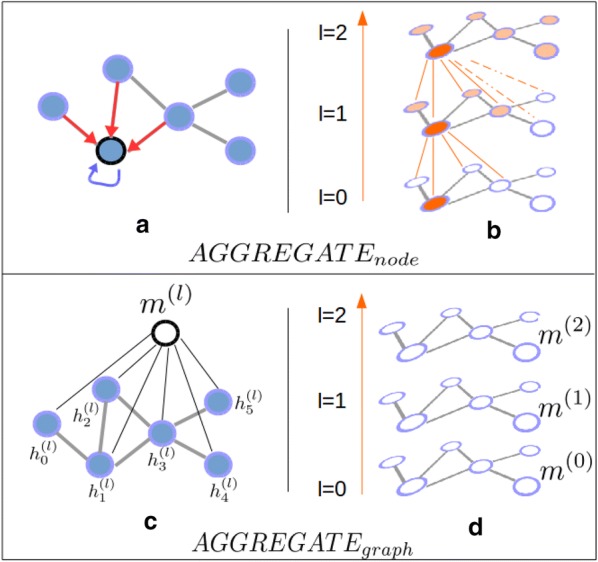
Sketch of the Graph neural network iterative process. (a) The $AGGREGATE^{(l)}_{node}$ function updates node representation vectors by aggregating information coming from itself and its neighbours. (b) As the process iterates, nodes receive information from further nodes in the graph. (c) The $AGGREGATE^{(l)}_{graph}$ function builds a graph-level representation vector by aggregating information from all nodes. (d) A graph-level representation is learned at each iteration

In more explicit words, at each layer *l*, all nodes aggregate information from their local neighbours in representation vectors $\mathbf {h}_i^{(l)}$, which are aggregated in an $\mathbf {m}^{(l)}$ representation of the molecule. At each iteration, nodes gain information from further nodes. These intuitions are illustrated in Fig. 2. Finally, a global representation $\mathbf {m}$ of the molecule is built by combining the $\mathbf {m}^{(l)}$ representations.

Various GNN methods can be considered, depending on the aggregation functions chosen at the nodes and graph levels, and on the $COMBINE_{graph}$ function that combines the $\mathbf {m}^{(l)}$ representations.

The flexibility and representation power of the GNN formulation in Alg. 1 rely on the aggregation functions. They update node-level representations based on the neighbourhood, and compute a graph-level representations (molecule representations, in our case) based on all nodes representations.

We will call the “minimal” formulation of GNN, the intuitive and simple encoding of the graph in which the $AGGREGATE^{(l)}_{graph}$ function is defined by the sum of all nodes representations, at each layer [28, 29, 32–35]:

1 $$\begin{aligned} \mathbf {m}^{(l)} = \sum _{i\in \mathcal {V}} \mathbf {h}_i^{(l)} \end{aligned}$$

This sum function captures in a single value, for each node feature, its distribution over all nodes.

We define the “minimal” $AGGREGATE^{(l+1)}_{node}$ function, the function that calculates the node representation $\mathbf {h}_i^{(l+1)}$ at layer $l+1$ based on the representations of its neighbourhood at layer *l*, after they are passed through a shared hidden layer. This function takes the form of Eq. 2:

2 $$\begin{aligned} \mathbf {h}_i^{(l+1)} = \sigma (\mathbf {W}_0^{(l)}\cdot \mathbf {h}_i^{(l)} + \sum _{j\in \mathcal {N}(i)}\alpha _{ij} \mathbf {W}_1^{(l)}\cdot \mathbf {h}_j^{(l)}) \end{aligned}$$

where, $\sigma$ refers to the sigmoid function, $\mathcal {N}(i)$ to the one-hop neighbourhood of node *i*, $\mathbf {W}_0^{(l)}$ and $\mathbf {W}_1^{(l)}$ are learnable weight matrices, and $\alpha _{ij}$ are learnable coefficients.

Finally, to provide a graph-level embedding $\mathbf {m}$ of the molecule, our “minimal” $COMBINE_{graph}$ function is simply the last layer representation $\mathbf {m}^{(L)}$, as in most studies.

The proposed chemogenomic neural network shown in Fig. 1 uses the minimal versions defined above for the $AGGREGATE^{(l)}_{node}$, $AGGREGATE^{(l)}_{graph}$, and $COMBINE_{graph}$ functions. Other more sophisticated versions of these functions have been proposed in other fields of graph representation learning. Interested readers will find details about these functions in  [31, 31, 36, 37] for $AGGREGATE^{(l)}_{graph}$ functions, in  [38–40] for $AGGREGATE^{(l)}_{node}$ functions, and in  [41] for $COMBINE_{graph}$ functions. We extensively tested these alternatives, but none of them improved the prediction performance of the proposed chemogenomic neural network (see Additional file 1), probably because molecular graphs are too small to benefit from these sophisticated embeddings that will not be further consider.

### Protein sequence encoder

Encoding a protein with a neural network encoder requires to define input attributes for amino acids such as the “one-hot” encoding, where amino acids are encoded by a binary vector containing a single one and 19 zeros (each bit corresponds to one of the 20 natural amino acids).

Since proteins are sequences of amino acids, their representations can be learnt by convolutional neural networks (CNN) and recurrent neural networks, in particular Long Short-Term Memory cells (LSTM) and bi-directional LSTM (bi-LSTM) as used in various prediction problems [42–47]. We tested CNN and bi-LSTM networks, but we observed that the bi-LSTM networks did not improve performance of DTI prediction (see Additional file 1). Therefore, we used a CNN to encode proteins.

### Combining protein and molecule encodings

In Fig. 1, the *Comb* block of the CN network that combines the learnt representations for proteins and molecules is simply the intuitive concatenation function. We investigated more sophisticated *Comb* blocks, but they did not improve the performance (see Additional file 1).

### The multi-layer perceptron $MLP_{pair}$

The chemogenomic neural network in Fig. 1 performs the DTI prediction task based on a final multi-layer perceptron (MLP), the most common deep neural network architecture. It consists of stacked fully connected layers, where each neuron at layer *l* takes as input all neuron outputs from layer $l-1$ and thus, directs its output to all neurons at layer $l+1$. The data are subjected to non-linear transformations across several layers, and intermediate layers can be viewed as hidden abstract representations of the original data. The $MLP_{pair}$ network can be viewed as a representation learning model, since it learns the best representations for the considered prediction task.

Overall, the chemogenomic neural network used throughout this paper is built with the minimal GNN defined above to encode molecules, the CNN to encode proteins, the concatenation *Comb* function, and a final $MLP_{pair}$ for the prediction task.

However, note that $MLP_{pair}$ can also directly use the concatenation of molecule and protein expert-driven representations as inputs, skipping the protein and molecule encoders, and the *Comb* block. This lead to state-of-the-art performances in many chemoinformatics applications, but was not tested yet in chemogenomics. Therefore, we also tested this approach, called FNN, as detailed in "Materials and methods".

## Related works

Direct predecessors of the proposed chemogenomic neural network are introduced below.

Ozturk et al. [48] used the following architecture for the four blocks of the chemogenomic neural network: (1) a three layers CNN on SMILES representations to encode molecules, (2) three layers CNN on protein sequences to encode proteins, (3) concatenation to combine the learnt molecule and protein encodings (4) a three layers feed-forward neural network to predict the affinity of molecules for proteins.

Their study was restricted to the kinases protein family, and therefore, was far from covering the protein diversity required to predict drugs unexpected targets. On this focused dataset, they obtained better or similar performance compared to Simboost [49] (a gradient boosting method) and KronRLS [12] (analogue of KronSVM but with kernelised Recursive Least Square) in terms of Mean Square Error (MSE). When “binarising” the outputs based on a threshold in pKd value to distinguish actives from inactives, their method performed slightly better than the considered shallow methods, in terms of AUPR. However, they did not compare to the simpler architecture of FNN with expert-driven descriptors for molecules and proteins as inputs, or to state-of-the-art shallow methods such as *NRLMF*.

In a more sophisticated approach, Tsubaki et al. [50] considered the following blocks: (1) GNN modules to encode molecules, in which the molecular graph-level representation $\mathbf {m}^{(l)}$ is learnt with an aggregation function $AGGREGATE^{(l)}_{graph}$ corresponding to the sum over the atom level representations, as in the “minimal” GNN defined above. (2) Protein encoding $\mathbf {h}_{prot}$ is performed with a CNN on amino acid representations which learns latent representations at the amino acid-level $\mathbf {h}_{(aa)_i}$. (3) The *Comb* module uses an attention mechanism that affects weights to learnt representations of each amino acid in the sequence. This allows interpretation of predictions based on which amino acids are involved in ligand binding. This deep learning method or a reference SVM-based method with a fingerprint-RBF kernel (not known to be state-of-the-art) was found to perform better, depending on settings, and on the considered metrics (precision, recall, or ROCAUC).

Although this study paved the way for deep learning in chemogenomics, going one step further than Ozturk et al. [48] in terms of deep learning architecture and technology, it suffers from several lacks in the discussion. First, the authors did not compare the prediction performances of their method to those obtained without the attention mechanism, which limits evaluation of their approach. Importantly, they did not provide a clear comparison with state-of-the-art shallow methods, and as in [50], they did not either compare to a simple FNN with expert-based descriptors as inputs, which often leads to the best prediction performances, as shown in "Results" section of the present paper.

Overall, the results of previous works in chemogenomics with deep learning are far from the striking gap in performance obtained by deep learning-based models in the image or natural language processing fields. They do not provide comparison to simpler deep learning methods, or to state-of-the-art shallow methods. Moreover, the datasets considered in these studies are very heterogeneous and often, do not cover the protein diversity required for drugs specificity prediction.

Deep learning approaches have also been proposed in QSAR methods, such as Koutsoukas et al.[51]. They can predict ligands for a given protein, or given bio-activity for molecules. When a training dataset of known ligands is available for a given protein, QSAR methods often display very good performances. However, these single-task methods cannot, as such, address the question of drug specificity. Importantly, they are not applicable to the orphan settings discussed in the present paper, which are key situations in drug specificity prediction problems.

## Materials and methods

### Datasets

The DrugBank database [52] is widely used as a bio-activity database. Although much smaller than PubChem [53] and ChEMBL [54], it contains around 17000 curated drug–target associations with high-quality standards. Therefore, we used the DrugBank database version 5.1.0 to build two interaction datasets that span proteins over the whole druggable proteome. The first dataset, called *DBHuman*, keeps interactions involving human proteins and their ligands, whereas the second, called *DBEColi*, keeps interactions involving *Escherichia coli* proteins and their ligands.

*DBEColi* is composed of 592 molecules targeting 314 proteins, and includes 874 interactions. *DBHuman* is composed of 4834 molecules targeting 2561 proteins, and includes 13,070 interactions. In both cases, known interactions are the positive training pairs, and most targets and drugs are involved in only one or two known interactions.

All other protein–ligand pairs are unlabelled. Most of them are expected not to interact, but a small number may be missing interactions. However, we considered unlabelled pairs as negative examples, as commonly assumed.

We also used a larger dataset derived from the ChEMBL database. We recovered all drugs (according to the filter defined in ChEMBL) targeting human proteins. Drugs had on average many more targets than in the DrugBank-based datasets, and we restricted the number of targets per drug to 40, to keep manageable computational times, leading to around 56,000 interactions between about 3700 small molecules and 1700 proteins. This dataset is used to evaluate whether the main conclusions drawn on the DrugBank-based datasets that we studied in more details, may generalise to other datasets.

To provide a source task for transfer learning, we also consider a PubChem-based dataset to pre-train the molecule encoder on auxiliary tasks. More precisely, we used the MolNet dataset collection [55] (namely, the PCBA dataset) to build a large dataset consisting in 439, 863 molecules and 90 binary classification tasks, i.e. PubChem bioassays. For each bioassay, molecules are labelled as active, inactive or unknown. When a molecule is labelled as unknown for a bioassay because it has not been experimentally tested, the prediction error for the corresponding task and molecule is set to zero.

### Evaluation procedures

We evaluated the performance by 5-fold nested cross-validation. Although we recorded the ROCAUC [56] and AUPR [57] scores for each test fold, we predominantly refer to the AUPR. Indeed, the AUPR score is considered as a more significant quality measure than the ROCAUC when negative interactions are in fact unknown interactions, as in DTI prediction, and when there are many more negative than positive samples in the test set, as in all experiments of this paper.

We considered four ways to split the two Drug-Bank-based datasets for the cross-validation scheme, corresponding to different settings. The $S_1$ setting corresponds to data split at random, $S_2$ to the orphan protein case (pairs in one fold only contain proteins absent in all other folds), $S_3$ to the orphan ligand case (pairs in one fold only contain molecules absent in all other folds), and $S_4$ to double orphan (pairs in one fold only contain proteins and molecules both absent in all other folds). This approach was suggested by Pahikkala et al. [58], because it allows assessment of performance in various real-life situations for chemogenomics in the context of drug specificity prediction. Note that the folds of $S_4$ were built by intersecting those of $S_2$ and $S_3$, so that there are 25 folds in the $S_4$ setting instead of 5.

In addition, we explored various “positive:negative” sample ratios in the test set (1:1, 1:2, 1:5 and 1:10).

### Reference shallow methods

As reference methods not involving deep learning, we considered the kronSVM [5] and *NRLMF* [23] approaches introduced in "Reference shallow methods in machine-learning for chemogenomics"section, because they led to state-of-the-art performances in various studies and datasets.

For kronSVM, the regularisation hyper-parameter of SVM, often called C, was optimised by assessing the performance in a 5-fold nested cross-validation scheme. We set the five hyper-parameters of *NRLMF* to their default value, as recommended in the original paper [23].

These two approaches require valid kernels for molecules and for proteins. For proteins, we used the local alignment kernel [59] (LAkernel) that mimics the Smith-Waterman (SW) score. LAkernel was shown to overtake other protein sequence kernels on protein homology detection [59], but it also proved relevant for drug virtual screening [25].

For molecules, we used the Tanimoto similarity measure, defined as the ratio between the number of substructures shared by the two molecular graphs over the total number of considered substructures. This similarity measure is very widely used in chemoinformatics, and it is also a valid kernel [60].

We also considered a Random Forest with proteochemometric descriptors, because this method lead to the best prediction performance on specific protein families datasets. More precisely, we used the implementation of Random Forest from scikit-learn [61] and considered 512-bits ECFP as molecular descriptors (extracted with the RDKit python package), and zscales(3) together with protein fingerprints(FP8) as protein descriptors (extracted with the R package), as suggested by van Westen et al. [62].

### Reference deep-learning method

As reference deep learning method, we considered a simple feed-forward neural network (FNN) with concatenation of expert-based numerical feature vectors as inputs for proteins and molecules, as mentioned in "Related works" section. To derive the expert-based descriptors for molecules, we extracted 1021-dimensional structural Morgan fingerprint vectors (analogues of ECFP4) with the RDKit library to extract the following features, based on Coley et al. work [35]: “atom identity”, “number of hydrogens”, “number of heavy neighbours”, “formal charge”, “is in ring”, “is aromatic”, “polar surface area”, “partial charge”, “chirality tag”. The chemical bond description contains: “bond type”, “is in ring”, “is aromatic”, “is conjugated”. For protein expert-based descriptors, we used the ProtR package to extract 1920-dimensional feature vectors corresponding to 11 protein level feature groups including amino acid composition (AAC), dipeptide composition (DC), autocorrelation descriptors, “composition, transition and distribution” descriptors (CTD), quasi-sequence-order descriptors (QSO), pseudo-amino acid composition descriptors (Pse-AAC), amphiphilic pseudo-amino acid composition descriptors (Am-Pse-AAC), topological descriptors at atomic level and total amino acid properties. Indeed, Ong et al. [63] reported that combining these descriptors provides the best results for the prediction of functional families with support vector machines.

The FNN comprises seven hyper-parameters: two architecture hyper-parameters (number of layers, number of neurons in each layer), three learning hyper-parameters (initial learning rate, batch size, learning rate decay factor), and two regularisation parameters (dropout probability and weight decay). The classical ranges of these parameters are given in the following in parenthesis. For the architecture hyper-parameters: number of layers ([1, 4]), number of neurons per layer ([10, 5000]). For learning hyper-parameters: batch size ([1, 100]), initial learning rate ($[10^{-5}, 10^{-2}]$) and learning rate decay factor ([0.8, 0.999]). For regularisation hyper-parameters: the probability of dropout ([0.0, 0.9]), and the weight decay ($[10^{-4}, 0.1]$). As commonly done for deep neural networks, we did not perform a full grid-search to optimise the seven hyper-parameters of the FNNs, because this would have required too much computational time. More precisely, we used a fixed train/validation/test data split. We started by optimising the learning hyper-parameters. The initial learning rate was optimised first (the other two parameters are set at median values in the above ranges), followed by the batch size, and the learning rate decay factor. This lead to an initial learning rate of $10^{-3}$, a batch size of 100, and a learning rate decay of 0.9. The architectures and regularisation parameters were optimised together, in such a way that the more the architecture was complex, the more the tested regularisation was strong (in our case, it meant that the regularisation parameters were high). Each of these four hyper-parameters was varied until the performance reached a plateau, where we stopped the search.

The optimised hyper-parameters were finally set to: 3 for the number of layers, 2000, 1000 and 100 for the successive three stacked fully connected layers, 0 (no regularisation) for the weight decay and dropout probability, and 0.9 for the learning rate decay. Overall, we observed that once the hyper-parameters reach a relevant value, the prediction performance does not vary significantly when the hyper-parameters remain in the same order of magnitude. Therefore, the above hyper-parameter values are good default values for this reference deep learning architecture.

### The proposed chemogenomic neural network

The chemogenomic neural network introduced in "Proposed chemogenomic neural network" section is represented in Fig. 1. The encoder for molecules is the “minimal” GNN, defined in "Proposed chemogenomic neural network" section by the Alg. 1, in which the $AGGREGATE^{(l)}_{graph}$ is the sum function, $AGGREGATE^{(l)}_{node}$ is defined in Eq. 2, and the $COMBINE^{graph}$ function used to build the molecule representation $\mathbf {m}$ is simply the last graph-level embedding $\mathbf {m}^{(L)}$. The GNN requires atom and bond features as input, which were calculated with the RDkit library, as for the reference FNN approach.

The protein sequence encoder is a stacked 1D convolutional neural layers (CNN), with amino acids described by their types in a one-hot encoding. The final representation learnt for the protein is the sum of the learnt amino acid representations resulting from the stacked convolutional layers.

Finally, the *Comb* operation in Fig. 1 is the concatenation of the molecule and protein learnt representations.

Compared to the architecture proposed by Tsubaki et al. [50], our chemogenomic neural network does not use bonds attributes for the molecule, and does not use any attention mechanism.

The chemogenomic neural network comprises three networks: the final $MLP_{pair}$ that makes the prediction (see Fig. 1), and the two protein and molecule encoders. The final $MLP_{pair}$ is formally identical to the FNN described in the previous section for the reference deep learning method. For the three networks, we optimised three types of hyper-parameters (architecture, learning, and regularisation hyper-parameters) by recording the performance on multiple train/validation/test data splits. It is computationally out of reach to perform a full grid search for all hyper-parameters, and therefore, each type of hyper-parameters was optimised separately. In the following, the ranges in which the hyper-parameters are explored are given in parenthesis. For the three networks, the learning hyper-parameters are the batch size ([1, 100]), initial learning rate ($[10^{-5},10^{-2}]$) and learning rate decay factor ([0.8, 0.99]). These hyper-parameters were optimised first, starting by the initial learning rate (the other two parameters are set at median values in the above ranges), followed by the batch size, and the learning rate decay factor.

The architecture hyper-parameters are the number of filters ([10, 1000]) and the number of layers ([2, 5]), both for the molecule and protein encoders, the convolutional filter size ([6, 12]) and the convolutional stride for the sequence encoder ([2, 6]). For the final $MLP_{pair}$, the architecture parameters are number of layers ([1, 4]), number of neurons per layer ([10, 5000]), as mentioned in the previous section for the reference FNN method. For the three types of networks, the regularisation hyper-parameters are the probability of dropout ([0., 0.9]) and the weight decay ($[10^{-4},0.1]$). We set the number of epochs to 100, and we also considered early stopping such that training stops if the performance (AUPR score) on the validation set does not increase in ten successive epochs. In practice, training was always stopped early.

Once the learning hyper-parameters were optimised, the architecture and the regularisation hyper-parameters were optimised together (except the convolutional filter size and stride of the protein encoder), in such a way that the more the architecture was complex, the more the tested regularisation was strong. The convolutional stride and the convolutional filter size of the protein encoder were optimised lastly, since they set the detection of amino acid motifs without changing the architecture complexity. Each of these hyper-parameters was varied until the performance reached a plateau, where we stopped the search.

This finally lead to set the number of filters to 100, the number of convolutional layers to 3, the stride to 3, the filter size to 8, the weight decay and dropout to 0, batch size to 20, the initial learning rate to $10^{-3}$, the learning rate decay factor to 0.9, and number of neurons in the last prediction layer to 100. We found that performance was not sensitive to relatively small variations of hyper-parameters, which means that a wide range of values for the hyper-parameters leads to the best performance. In particular, adding a small quantity of dropout (until 0.5) leads progressively to a longer training and to a loss of training performance, whereas it did not significantly improve the performance on the validation data.

Overall, these hyper-parameters values are good default values for the proposed chemogenomic neural network architecture.

## Results

### Comparison of the proposed chemogenomic neural network to reference methods

We first compared the performance of the chemogenomic neural network (CN) to those of state-of-the-art reference methods, namely kronSVM and *NRLMF* for machine-learning shallow methods, and FNN with expert-based descriptors as inputs for deep-learning methods, as described in "Materials and methods". All performances are reported in Supporting Materials, displayed in the format “mean score±score standard deviation”, for various ratios of positive:negative test samples.

**Fig. 3:**
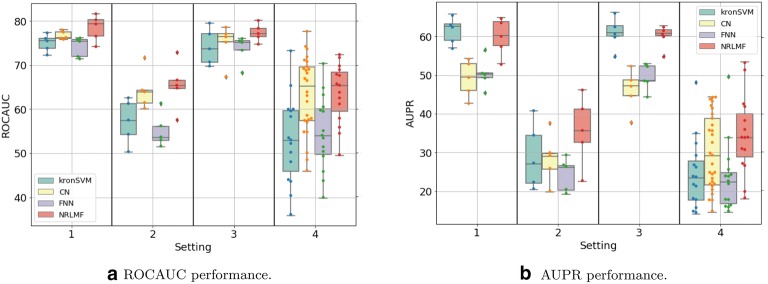
Performances on *DBEColi* for the *S*1 (random split), *S*2 (orphan proteins in test set), *S*3 (orphan molecules in test set), and *S*4 (double orphan in test set) settings, with a positive:negative samples ratio set to 1:5. For each setting, the order from left to right in which the results are displayed is given in the legend

**Fig. 4:**
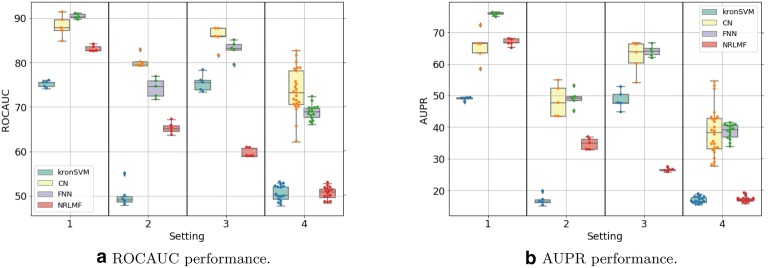
Performances on *DBHuman* for the *S*1 (random split), *S*2 (orphan proteins in test set), *S*3 (orphan molecules in test set), and *S*4 (double orphan in test set) settings, with a positive:negative samples ratio set to 1:5. For each setting, the order from left to right in which the results are displayed is given in the legend

Figures 3a,  b, 4a, b display the ROCAUC and AUPR performance obtained on the *DBEColi* and *DBHuman* datasets, for a “positive:negative” test sample ratio set to 1:5. The AUPR and ROC-AUC scores obtained for other ratios are gathered in Additional file 2.

First, we observe that the best performing method depends on the dataset and on the setting.

On the small *DBEColi* dataset (874 interactions), ROCAUC scores are very close for all methods, but the shallow *NRLMF* method tends to perform the best overall. For the more important AUPR score (as explained in "Materials and methods" section), the *NRLMF* and kronSVM shallow methods clearly outperform deep methods. Among deep learning methods, overall, the proposed CN performs better than the FNN reference method. Our results show that, on small datasets (a few thousand interactions, or less), shallow methods should be preferred. *NRLMF*  appears as a good default method, although its performances in the $S_2$ and $S_4$ orphan settings are very close to those of the proposed CN deep method, because it is simpler and computationally efficient.

On the larger *DBHuman* dataset (13,070 interactions), overall, the two deep learning methods CN and FNN outperform the two shallow methods *NRLMF* and kronSVM, both in terms of ROCAUC and AUPR. This is particularly true in the three orphan settings ($S_2$, $S_3$, and $S_4$) which are important in the context of drug specificity prediction. The proposed CN deep learning approach globally performs sightly better than the FNN reference deep learning approach in ROCAUC, and similarly in AUPR. Overall, for large datasets (in the range of ten thousand of interactions, or above), deep learning methods should be preferred to shallow methods, because they perform better on a wide panel of settings. This tendency will be confirmed below in the Discussion section, on the larger ChEMBL-derived dataset (56,000 interactions).

Globally, the performance reached on the *DBHuman* are 10 points higher than those reached on *DBEColi*, which was expected since there are much more data, i.e. more information to train the models, in *DBHuman* than in *DBEColi*.

Regarding the settings, the best scores are obtained for $S_1$, the worst for $S_4$, and the scores obtained for $S_2$ and $S_3$ are intermediate. Indeed, $S_4$ corresponds to the double orphan test set, where no pair in the training set contains proteins or ligands present in the tested pairs to guide the predictions, whereas the models can rely on training pairs containing either the same proteins in $S_2$ or the same ligands in $S_3$. The loss of performance between the random ($S_1$) and the double orphan ($S_4$) settings is about 10 points of ROCAUC and 20 points of AUPR. More importantly, the prediction on $S_4$ varies more depending on the test folds than in the other settings. This can be understood as a consequence of the small size of the test set in the $S_4$ setting (any test fold is 1/25*th* of the total amount of data whereas they represent 1/5*th* in the other settings), which might result in more heterogeneous test sets. Interestingly, the performance reached by all models is better for $S_3$ than for $S_2$, which suggests that predicting protein targets for new molecules is a more difficult task than predicting ligands for new proteins, as already observed in previous studies [25, 58].

Let us conclude by a few remarks. We considered (1:1, 1:2, 1:5 and 1:10) “positive:negative” samples ratios in the train set. We did not test larger proportions of negatives because it would be hardly computationally tractable, and it would correspond to strongly imbalance datasets.

The kronSVM and *NRLMF* shallow methods did not benefit from an increasing number of negative samples in the train set, whereas deep learning-based methods strongly did between 1:1 and 1:5. More precisely, increasing the number of negative samples in the training set, up to five times the number of positive samples, increased the performance of CN and FNN by about 10% in AUPR and 2% in ROCAUC for the *DBHuman* dataset, and by 20% in AUPR and 10% in ROCAUC for the smaller *DBEColi* dataset. We also observed that CN benefits slightly less than FNN from an increasing number of negatives in the training set. The performance of CN and FNN did increase between a ratio of 1:5 and 1:10 only in some cases, meaning that the benefit reaches a plateau.

We did not correct the imbalance proportions of labels in the training set for deep learning-based approaches, because it lead to similar performances, or lower by few percent. Surprisingly, we never observed an improvement in performance when correcting positive/negative samples imbalance for deep learning-based approaches, which we cannot explain easily.

### Evaluation of the combination of expert-based and learnt features

**Fig. 5:**
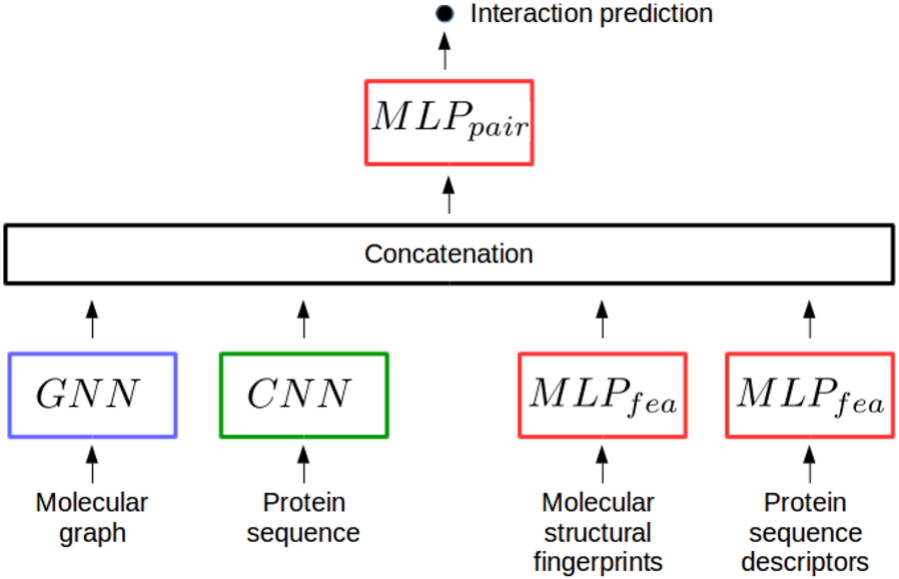
Chemogenomic neural networks combining expert-based and learnt features in one step

**Fig. 6:**
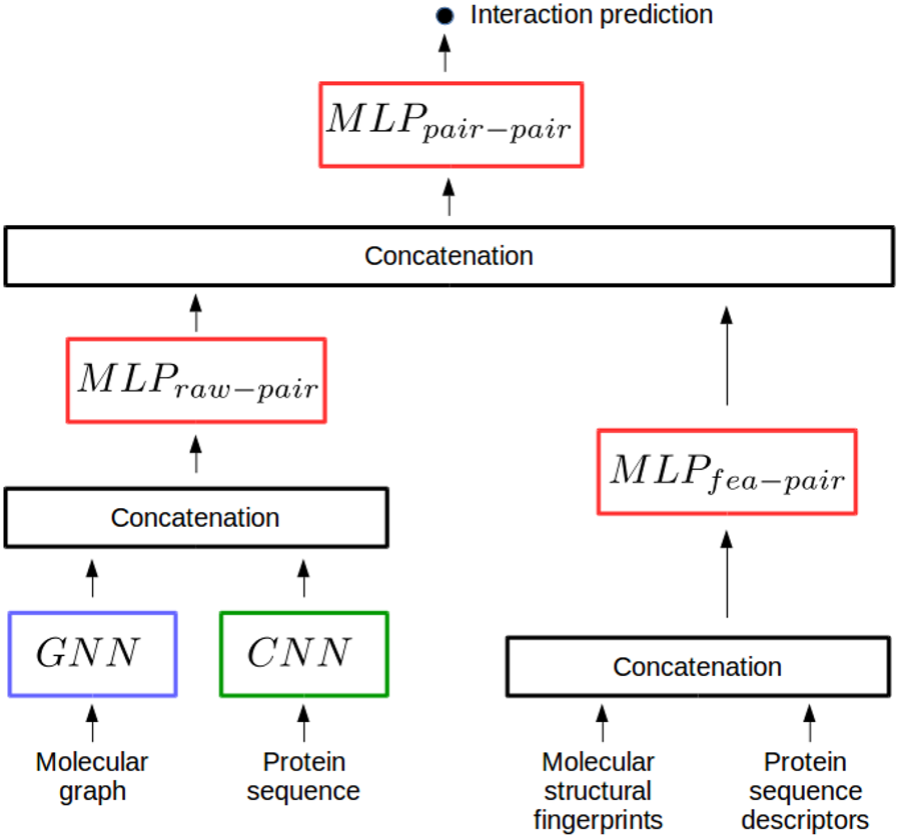
Modification of the proposed chemogenomic neural network combining expert-based and learnt features

We showed that the reference FNN directly trained with expert-based protein and molecule features as inputs, outperforms the proposed chemogenomic neural network in some settings. Therefore, we modified the architecture represented in Fig. 1, and considered two architectures that integrate these expert-based and learnt features for proteins and molecules into a final pairwise representation, as displayed in Figs 5 and 6. The idea was to test whether the proposed CN approach could benefit from the two forms of representations.

More precisely, the architecture in Fig. 5 combines four types of representations in one step: (i) the molecular graph, (ii) the expert-based molecule features, (iii) the protein sequence and (iv) the expert-based protein features. For the multi-layer perceptrons $MLP_{fea}$ and $MLP_{pair}$, we explored the hyper-parameter spaces by varying the number of layers (from 1 to 3), the number of hidden units (in {10,100,200,1000,2000}) while promoting pyramidal architectures, as motivated in [64], and the quantity of dropout (in {0,0.3,0.6,0.9}).

To rule out the possibility of an implementation error, we performed two sanity checks. First, we checked that we recover the performance obtained by the simple reference FNN with expert-based features as inputs, when we silent the protein sequence and molecular graph encoders. Similarly, we checked that we recover the performance obtained with expert-based features by the proposed CN method, when silencing the $MLP_{fea}$ and $MLP_{pair}$ networks.

The performance reached by the model architecture in Fig. 5 is significantly lower than that obtained by the CN chemogenomic neural network of Fig. 1, and by the reference FNN with expert-based features, on the four $S_1$, $S_2$, $S_3$, $S_4$ settings of the *DBEColi* dataset (data not shown). A possible explanation is that simultaneous training of the $MLP_{fea}$ and $MLP_{pair}$ networks with molecular graph and protein sequence encoders is an ill-conditioned non-convex optimisation problem.

To address this issue, we separately pre-trained the pairs of neural networks encoding expert-based and learnt features, before re-training them together on the same training data. More precisely, the final $MLP_{pair}$ network is trained with the GNN and CNN networks alone, then with the $MLP_{fea}$ for proteins and molecules alone. The pre-trained GNN and CNN, and $MLP_{fea}$ networks are finally re-trained together, as in the architecture displayed in Fig. 5. The parameter starting values of protein and molecule networks are those optimised in the pre-training steps, whereas the starting values of parameters in the $MLP_{pair}$ are randomly initialised. This procedure allowed retrieve, but not overtake, the performance reached by an FNN with only expert-based representation as inputs, even when increasing the width and depth of $MLP_{pair}$.

To further explore joint optimisation of neural modules for expert-based and learnt features extraction, we considered the architecture in Fig. 6. It combines the pairwise expert-based and learnt representations through a single relatively small layer. Typically, $MLP_{pair\text {-}pair}$ is composed of a single hidden layer of 50 neurons. In the following, we call this model the $CN\text {-}feaMLP$ approach. We varied the neural architecture and dropout probability of $MLP_{fea\text {-}pair}$ similarly as for $MLP_{fea}$, and those of $MLP_{raw\text {-}pair}$ and $MLP_{pair\text {-}pair}$ similarly as for $MLP_{pair}$ in the architecture displayed in Fig. 5.

The performances of this network for a single test/train split are reported in Table 1 at the row named $CN\text {-}feaMLP$. Remarkably, this approach outperformed the CN chemogenomic neural network on the $S_1$ setting, while performing similarly or above on the three others.

**Table 1:** AUPR score for the two transfer learning modifications of the chemogenomic neural network CN, based on a single train/validation/test split of the *DBEColi* dataset

	Raw ($S_1$)	Orphan proteins ($S_2$)	Orphan molecules ($S_3$)	Double orphan ($S_4$)
Chemogenomic neural network (CN)	$39.57 \pm 4.17$	$26.74 \pm 2.49$	$43.74 \pm 2.35$	$\mathit{24.63} \pm \mathit{1.89}$
Curriculum learning	$45.06 \pm 2.64$	$21.43 \pm 3.62$	$\mathit{51.45} \pm \mathit{3.03}$	$20.97 \pm 2.70$
$CN-feaMLP$	$\mathit{50.616} \pm \mathit{2.71}$	$\mathit{30.89} \pm \mathit{4.93}$	$42.04 \pm 2.95$	$\mathit{25.77} \pm \mathit{3.60}$

The curriculum learning line corresponds to pre-training the molecule encoder of CN on the *DBHuman* dataset. The standard deviations are obtained by repeating 5 times the evaluation procedure

**Fig. 7:**
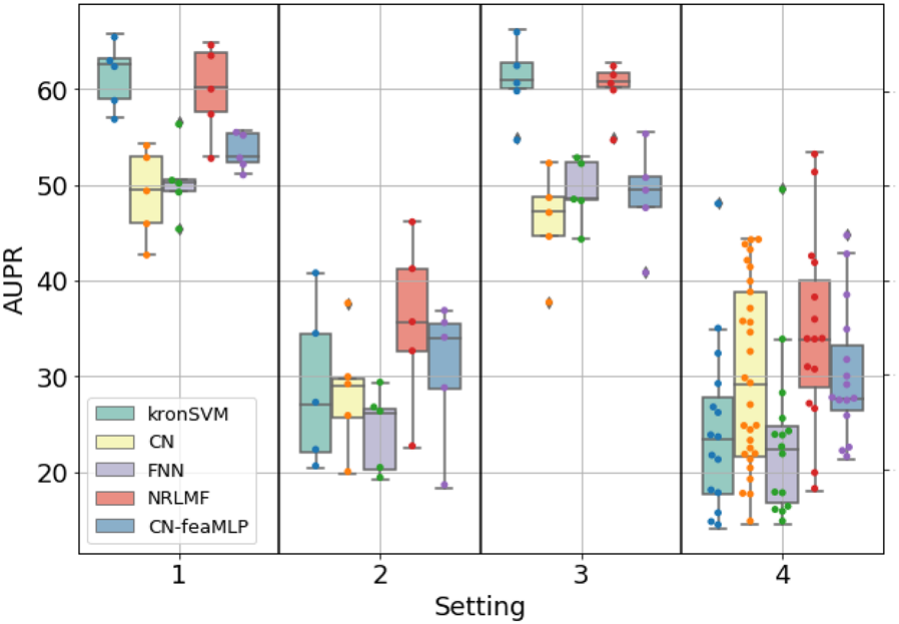
AUPR scores obtained via 5-fold nested cross-validation on the *DBEColi* dataset for the *S*1 (random split), *S*2 (orphan protein in test set), *S*3 (orphan molecule in test set), and *S*4 (double orphan) settings. The performances of $CN\text {-}feaMLP$ are compared to the reference shallow methods and to FNN and CN as reference methods for deep learning. For each setting, the order from left to right in which the results are displayed is indicated in legend

**Fig. 8:**
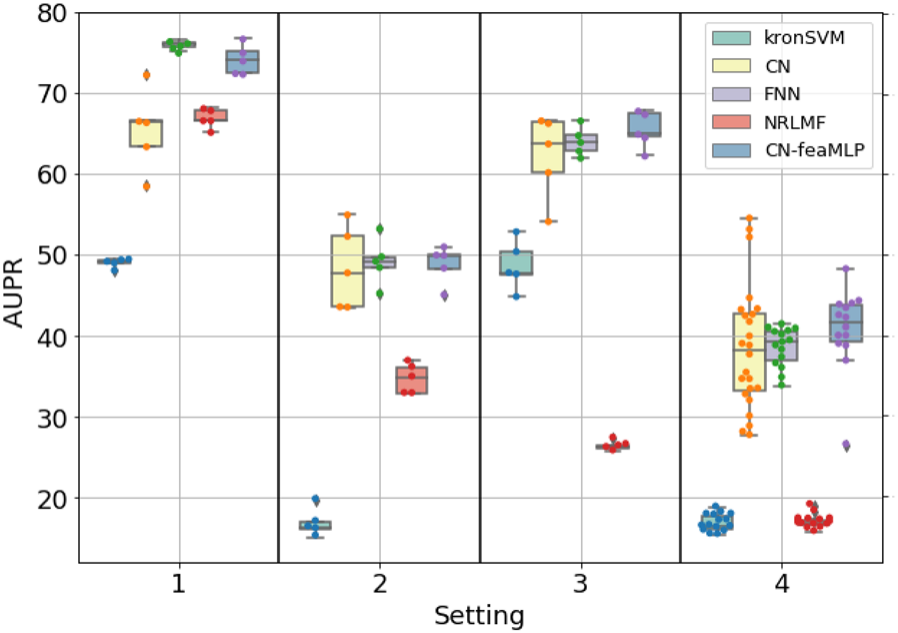
AUPR scores obtained via 5-fold nested cross-validation on the *DBHuman* dataset for the *S*1 (random split), *S*2 (orphan proteins in test set), *S*3 (orphan molecules in test set), and *S*4 (double orphan) settings. The performances of $CN\text {-}feaMLP$ are compared to the reference shallow methods and to FNN and CN as reference methods for deep learning. For each setting, the order from left to right in which the results are displayed is indicated in legend

To evaluate this approach in more details, we assessed its performance on the $S_1$, $S_2$, $S_3$ and $S_4$ settings using a 5-fold nested cross-validation scheme, with a test and train “positive:negative” ratio of 1:5 (results of Table 1 corresponded to a single train/test split). The performance on *DBEColi* and *DBHuman* are reported in Figs. 7 and 8, together with those of reference methods and of the CN chemogenomic network represented in Fig. 1. For the *DBEColi* dataset, the $CN\text {-}feaMLP$ model outperforms both the CN and the FNN reference model on $S_1$ and $S_2$, and performs similarly, yet slightly better, on $S_3$ and $S_4$. However, on this small dataset, the performances remain lower than those of the *NRLMF*  shallow method, particularly on $S_1$ and $S_3$.

On the larger *DBHuman* dataset, the $CN\text {-}feaMLP$ model outperforms the CN chemogenomic neural network in the $S_1$ setting, but it does not overtake the FNN approach. In the $S_2$, $S_3$ and $S_4$ orphan settings, the $CN\text {-}feaMLP$ approach reaches slightly better performances than both the CN chemogenomic neural network and the reference FNN method. Overall, on this larger dataset, the two shallow models, kronSVM and *NRLMF*, still have much lower performances than the deep learning models, and the proposed $CN\text {-}feaMLP$ model displayed robust performances that remained among the best achieved in the four settings.

We conclude that data augmentation techniques based on multi-views of the data improve the prediction performance of the deep-learning algorithm CN. For relatively large datasets like *DBHuman*, that are widely available today, the $CN\text {-}feaMLP$ model is good default method that robustly outperforms or competes with reference shallow or deep learning methods, over the four considered settings. We believe that integrating other multiple description views of molecules and proteins within the same model, is an interesting direction for future developments. Importantly, the CN chemogenomic network provides a versatile architecture to incorporate such views.

In line with this idea, we now explore transfer learning as an implicit data augmentation approach, and explore its interest to enhance the performance of the proposed chemogenomic neural network CN. Indeed, transfer learning combines different tasks related to the same types of data (proteins or molecules in our case), with the aim of providing additional information or representation power brought by different prediction tasks.

### Evaluation of transfer learning for chemogenomics

#### Principles of transfer learning in chemogenomics

The principle of transfer learning is to gain knowledge by solving one problem and apply this knowledge to a different but related problem. Transfer learning can take two forms: pre-training, or co-training with other prediction tasks (even unsupervised tasks) for which large datasets are available. This may allow sharing of information and improve the performances of the chemogenomics task.

Co-training refers to a strategy in which several prediction tasks are trained simultaneously, while sharing parameters of the neural networks, in order to learn a richer set of features. We did not consider co-training, because this approach suffers from lack of flexibility in training, as observed in the field of Natural Language Processing [65].

In pre-training, also called “curriculum learning”, a model is first trained on a task for which a large dataset is available, called the source task. Then, the pre-trained model is fine-tuned by re-training the weights on the task of interest, called the target task, for which a smaller training set is available. Therefore, when training on the target task, the initial parameters of the encoder are not randomly chosen, but are assigned to their optimised values according to the source task.

Paul et al [66] explored transfer learning in a chemoinformatics problem related to organic solar cell screening, but this task only screened chemicals, not (protein,molecule) pairs, as in the present study.

**Fig. 9:**
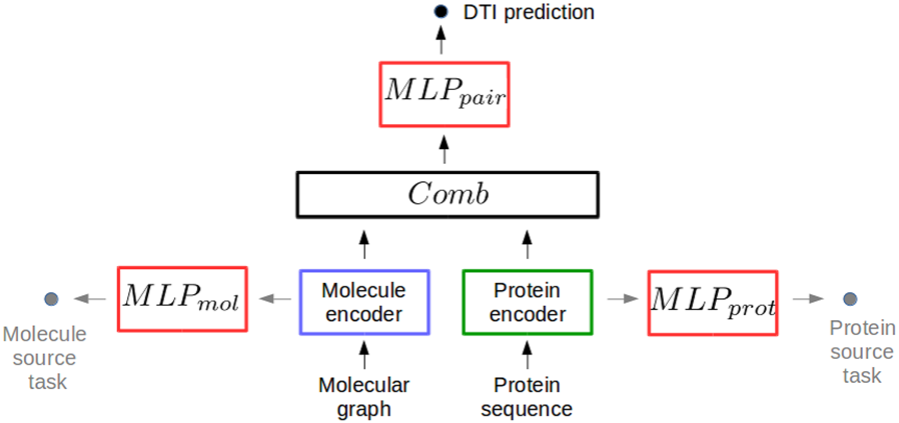
Sketch of the chemogenomic neural network CN of Fig. 1, modified for curriculum learning

Fig. 9 illustrates the principle of curriculum learning, in the case of a DTI prediction task with the chemogenomic neural network CN.

First, the protein sequence and molecular graph encoders are trained with molecule- and protein-specific source tasks, corresponding to the grey path in Fig. 9. Then, the pre-trained encoders are re-trained, while training the $MLP_{pair}$ chemogenomic prediction task corresponding to the black path in Fig. 9.

Alternatively, it is possible to “freeze”, i.e. not retrain, the pre-trained molecule and protein encoders, but only train the final $MLP_{pair}$ with the representations learnt from the pre-trained encoders as inputs. If the source and target datasets are related “enough”, this can improve predictions on the target task.

In the next sections, we explore two pre-training settings: one with a formally identical source task, and another with a different source task.

#### Transfer learning by pre-training with a formally identical source task

The results presented in the previous sections showed that our CN chemogenomic network could outperform reference deep and shallow machine-learning methods (kronSVM or *NRLMF* ) on large datasets like *DBHuman*, but not on small datasets like *DBEColi*. Therefore, we chose *DBEColi* to explore whether transfer learning by pre-training on a larger and formally identical task, i.e. prediction on the *DBHuman* dataset, can improve the prediction performance of CN on small datasets like *DBEColi* to reach those of shallow methods.

This setting is slightly different from that in Fig. 9, because the source and target tasks are formally identical: the source task of DTIs prediction on *DBHuman* is used to pre-train the whole CN network (protein encoder, molecule encoder and $MLP_{pair}$), before re-training the whole CN on the smaller *DBEColi* dataset.

Before discussing our results, let us start with general observations about implementation of curriculum learning with the chemogenomic neural network CN.

First, we performed a sanity check to challenge our implementation of curriculum learning: we checked that re-training the pre-trained model immediately leads to the best performance.

Second, we found that curriculum learning often allowed to reduce the number of training epochs for training the $MLP_{pair}$ network in the second phase. This illustrates that pre-trained molecule and protein encoders are closer to the optimal solution than encoders whose weights are randomly initialised.

Third, we pre-trained and loaded per block the three sub-networks of the chemogenomic neural network: (i) the protein sequence encoder, (ii) the molecular graph encoder, and (iii) the final prediction $MLP_{pair}$ network. We varied the number of layers and neurons in the $MLP_{pair}$ network, in order to search for the best architecture when freezing one or the two encoders. Indeed, a fully connected neural network like $MLP_{pair}$ may need a relatively large width and depth to leverage the representations extracted by pre-trained frozen encoders.

We pre-trained the three parts of the chemogenomic neural network (the protein sequence encoder, the molecular graph encoder and the prediction $MLP_{pair}$ network) on the larger *DBHuman* dataset before re-training on the *DBEColi* dataset. Interestingly, this led to a loss of performance on $S_1$, with 0.2 in AUPR score instead of 0.4. This resulted from the protein encoder pre-trained on *DBHuman*. Indeed, pre-training on *DBHuman* and freezing the protein sequence encoder, before training the molecular graph encoder and the $MLP_{pair}$ directly on *DBEColi* with randomly initialised parameters, led to the same decrease in AUPR score. On the contrary, pre-training and freezing the molecular graph encoder with *DBHuman* before training the protein sequence encoder and the prediction $MLP_{pair}$ directly on *DBEColi* with randomly initialised parameters, improved the performance in some settings with respect to CN directly trained on *DBEColi*, as shown in Table 1 at the row called “curriculum learning”. More precisely, for the $S_1$ (random splits) and $S_3$ (orphan molecule) settings, which are expected to benefit from a better trained molecular encoder, pre-training on *DBHuman* leads to significant improvement in performance. This is not the case of the $S_2$ (orphan proteins) which would have required pre-training of the protein encoder, and for the most difficult $S_4$ (double orphan) setting.

In fact, although the source and target tasks are formally identical, *DBEColi* contains E. coli proteins and their ligands, whereas *DBHuman* contains human proteins and their ligands. Therefore, the two datasets may present statistical bias in protein sequences characteristics, since the distribution of protein sequences in bacteria is expected to differ from that of human proteins. However, both datasets contain more homogeneous small organic drug-like molecules. This may explain a loss of performance when pre-training with human proteins, while pre-training only the molecular graph encoder on the *DBHuman* dataset improves DTIs prediction in the relevant settings ($S_1$ and $S_3$).

Overall, these results show that transfer learning between two formally identical tasks can be observed with the chemogenomic neural network CN. Improvements were only observed here when the molecular graph encoder is pre-trained, due to bias between proteins in the source and the target datasets. However, pre-training the protein encoder on a larger dataset involving *E. coli* protein interactions may further improve the prediction performance of CN.

#### Transfer learning by pre-training with different source tasks

To further investigate the interest of transfer learning with the CN chemogenomics deep learning algorithm, we now compare the prediction performance obtained on the small target task *DBEColi*, when the large source task is of different although related nature. We used the PCBA dataset as source task. It contains information about 90 bio-activities for hundreds of thousands of molecules (see "Materials and methods"). PCBA appeared relevant for pre-training the molecule encoder of the chemogenomic network, because the bio-activities of molecules result, at least in part, from their overall interaction profile with proteins in the cell. More precisely, the source task used to pre-train the molecule encoder consists in simultaneous predictions of molecules activities in 90 bioassays.

We evaluated pre-training of the molecular graph encoder on *PCBA* by 5-fold nested cross-validation with a test and train “positive:negative” ratio of 1:5, in the four $S_1$, $S_2$, $S_3$ and $S_4$ settings of the *DBEColi* dataset, and compared it to pre-training the molecular graph encoder on *DBHuman*. In the case of pre-traning with *DBHuman*, only the parameters of the molecule encoder are kept as initial values for re-training on *DBEColi*, since we showed that pre-trained parameters as initial values for the protein encoder decreased performances. Therefore, parameters of the protein encoder and of the $MLP_{pair}$ networks were randomly initialised. In the case of pre-training with *PCBA*, the source task is to predict the PubChem assay bio-activities. The pre-training step again only involves the molecule encoder and the $MLP_{pair}$, since this dataset only contain bio-activities for molecules. Then, the parameters of the molecule encoder are kept for re-training the chemogenomic network on *DBEColi*, while the protein encoder is randomly initialized.

**Fig. 10:**
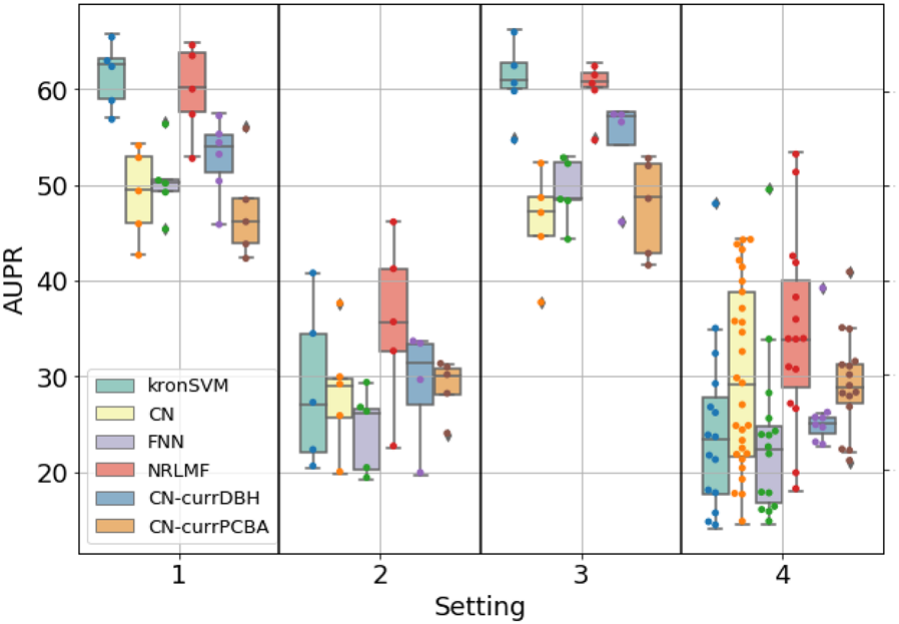
AUPR scores obtained with a 5-fold nested cross-validation scheme for the $S_1$ (random split), $S_2$ (orphan protein in test set), $S_3$ (orphan molecule in test set), and $S_4$ (double orphan) settings on the *DBEColi* dataset. The performance of $CN\text {-}currPCBA$ and $CN\text {-}currDBHuman$ are compared to the reference shallow methods (kronSVM and *NRLMF*) and to FNN and CN as reference methods for deep learning. For each setting, the order from left to right in which the results are displayed is indicated in legend

The performances are reported in Fig. 10 with those of the reference methods. The final model obtained when pre-training the molecular graph encoder on the *PCBA* (resp. *DBHuman*) dataset is called $CN\text {-}currPCBA$ (resp. $CN\text {-}currDBHuman$) in Fig.10

Overall, pre-training the molecular graph encoder on *DBHuman* improved the prediction performance of the chemogenomic network in the S1 (random splits) and S3 (orphan molecules) settings, with respect to direct training on *DBEColi* (CN and $CN\text {-}currDBHuman$ in Fig.10). This confirmed in 5-fold nested cross validation the observations made in the previous section on a single train/validation/test split: transfer learning can be observed between these two tasks in the CN chemogenomic deep learning method.

Pre-training the molecular graph encoder on *PCBA* yielded to similar performance than direct training on *DBEColi* for the four settings, but did not allow to reach the performance observed when pre-training with *DBHuman* ($CN\text {-}currPCBA$ in Fig. 10).

This shows that the nature of the source task has an impact on the efficiency of knowledge transfer. It illustrates a rather intuitive idea that, knowledge transfer from a smaller more similar the source task (*DBHuman*) may better improve the prediction performance of the target task, than knowledge transfer from a larger but less similar source task (*PCBA*). However, it denies the growing paradigm in artificial intelligence that when providing algorithms with large amounts of diverse data (or so-called “big data”), even if these data are somewhat loosely related to the tasks of interest, the algorithms will “find their way” to learn by extracting the relevant information.

However, Fig. 10 shows that, in general, the reference shallow methods kronSVM or *NRLMF* still led to the best performance on this small *DBEColi* dataset, and even when transfer learning occurs for deep learning methods, at least for the source datasets used in the present study.

## Discussion and conclusion

Our goal was explore shallow and deep learning methods for proteome-wide prediction of DTI, in order to point at a few key experiments to perform and avoid costly failure later in the drug development process, due to unacceptable side-effects.

One important aspect of the present work is that we discuss a few key aspects for drug specificity prediction:

1. Our study builds on and provides new insights on the chemogenomic neural network initially proposed by several authors [48, 50, 67], in which the methods are not compared to state-of-the-art shallow and deep learning methods.
2. we considered four settings, $S_1$ (random splits), $S_2$ (orphan proteins), $S_3$ (orphan molecules), $S_4$ (double orphan), in order to compare the performance of the methods in proteome-wide search of off-targets for a new drug, a problem involving many predictions that are closer to the orphan settings $S_2$, $S_3$, or $S_4$ than to the *S*1 setting for which high performance are reached by all methods.
3. We also studied the performance of the methods on datasets of various sizes. Indeed, although large DTIs datasets are becoming available nowadays, learning on a small but well focused dataset with respect to the problem at hand might sometimes be a better strategy. Therefore, it is interesting to assess prediction performances of the chemogenomic methods both on large and small datasets.

For large datasets like *DBHuman* (in the range of 10,000 interactions and above), we showed that the proposed deep learning chemogenomic neural network CN outperforms state-of-the art reference shallow machine-learning methods, particularly in the important $S_2$, $S_3$ and $S_4$ orphan settings, and competes with the reference deep learning FNN method that uses expert-driven descriptors as inputs.

In order to evaluate whether this result would generalise to other large datasets, we ran the CN deep learning method and the *NRLMF*  shallow method on another large dataset built from the ChEMBL database (see "Materials and methods"). The results in the $S_1$, $S_2$, $S_3$ and $S_4$ settings, for a single train/validation/test split are displayed in Table 2. They show that the CN method outperforms the shallow *NRLMF*  method in two of the three key orphan settings ($S_3$ and $S_4$), while performing similarly in $S_1$ and $S_2$. Although we did not conduct a full study on this larger dataset (56,000 interactions) for computational reasons, the results in Table 2 show the same trends as those obtained on the DrugBank-based *DBHuman* dataset: deep learning methods should be used on large datasets (in the range of 10,000 interactions or more).

**Table 2:** AUPR scores on the Chembl-based dataset in the four $S_1$, $S_2$, $S_3$, or $S_4$ settings, and for a test sample positive:negative ratio 1:5

	$S_1$	$S_2$	$S_3$	$S_4$
NRLMF	$77.95\pm 0.27$	$58.85\pm 5.74$	$58.71\pm 1.3$	$30.6\pm 1.54$
CN	$76.91\pm 2.15$	$55.44\pm 6.26$	$68.89\pm 7.41$	$35.61\pm 3.01$

In particular, our study highlights that, for large datasets, the FNN algorithm with expert-based protein and molecule descriptors appears as a simple deep learning approach that provides state-of-the-art performance in many settings. It should be considered as a good default method, and as a reference method for future chemogenomic benchmark studies. In particular, we did not combine sophisticated expert-crafted descriptors, as proposed in other studies with shallow algorithms [68–71], which leaves space for improvement and reinforces the interest of this approach.

However, the proposed chemogenomic network CN offers a versatile architecture for more sophisticated strategies, such as data augmentation approaches or transfer learning, in order to enhance the representation power of protein or molecule encodings with respect to the problem at hand.

For smaller datasets like *DBColi* (less than a few thousands of interactions), we observed that *NRLMF* shallow method provides the best performance for DTIs prediction. It is a good default method for large scale DTI prediction, and provides a reference method for future chemogenomics benchmark studies on small datasets.

To further explore this point, we also considered the Random Forest algorithm with proteochemometric descriptors proposed in van Westen et al. [8], because they displayed the best performances on small datasets in specific families of proteins (i.e. not in the chemogenomics setting considered in the present paper). We tested the descriptors recommended in this study (z-scales(3) together with protein fingerprints(FP8) as protein descriptors, and ECFP as molecule descriptors) on the *DBColi* dataset. The prediction performances of this method are shown in Table 3, where we also recall those of the other methods tested in the present paper. Random Forests with proteochemometric descriptors did not reach the performance of the other shallow methods, but they overtook those of FNN and CN deep learning methods. However, we are aware that the prediction performances depend on the dataset, and that methods may rank differently on family-focused datasets used in [8], and on more diverse datasets *DBColi* dataset.

**Table 3:** AUPR scores (mean and standard deviation obtained by nested 5-fold cross-validation) on the *DBEColi* dataset in the four $S_1$, $S_2$, $S_3$, or $S_4$ settings, and for a test sample positive:negative ratio 1:5

	$S_1$	$S_2$	$S_3$	$S_4$
kronSVM	$\mathit{61.55} \pm \mathit{3.11}$	$\mathit{28.95} \pm \mathit{7.66}$	$\mathit{60.96} \pm \mathit{3.73}$	$24.4 \pm 8.8$
*NRLMF*	$\mathit{59.89} \pm \mathit{4.35}$	$\mathit{35.62} \pm \mathit{8.07}$	$\mathit{60.06} \pm \mathit{2.73}$	$\mathit{34.5} \pm \mathit{9.75}$
FNN	$51.55 \pm 2.55$	$24.26 \pm 3.91$	$49.34 \pm 3.11$	$22.97 \pm 6.59$
Chemogenomic neural network (CN)	$49.08 \pm 4.31$	$\mathit{28.38} \pm \mathit{5.81}$	$46.14 \pm 4.92$	$\mathit{27.0} \pm \mathit{6.96}$
RF with proteochemometric features	$53.49 \pm 2.19$	$23.6 \pm 4.13$	$51.16 \pm 4.32$	$22.51 \pm 5.36$

All methods were trained on drug-like molecules and drugabble proteins, because the aim was drug specificity prediction. However, all tested methods readily apply to prediction of interactions between other types of molecules or proteins, because only the protein sequences and molecule structures are required. However, the methods should be re-trained, because the good default hyper-parameters provided in the present study might not be optimal for highly different datasets (for example, not druglike molecules, or very different proteins such as plant proteins). This point is particularly critical for the CN and FNN deep learning methods, because they contain more hyper-parameters than shallow methods. In addition, the results of the present study might not extrapolate to datasets highly different from those considered here, because methods might rank in different orders on very different datasets.

An important contribution of the present paper is to explore data augmentation techniques in deep learning for chemogenomics. We investigated two directions for integration of different data sources:

1. Learning with multiple views of the data: combining learnt and expert-driven features in the CN network yielded to substantial improvements, and future efforts could be devoted to the design of neural networks that integrate multiple views of molecules and proteins within the same model. However, in the case of small datasets like *DBEColi*, these performance improvements did not allow to reach those of shallow machine-learning methods.
2. Transfer learning with larger similar or with different source tasks. We report that curriculum learning may also improve the prediction performance if the source task is highly similar to the target task.

Overall a promising direction to improve the proposed chemogenomic neural network CN would be to integrate various data views and use large similar auxiliary tasks, in order to leverage relevant chemical information such as bioassay signatures and drug-induced phenotypes that are available in many public databases.

## Supplementary information

**Additional file 1.** Tables presenting the ROCAUC and AUPR scores on the *DBH* and *DBEC* datasets for various positive:negative ratios.

**Additional file 2.** Principles and results of several promising methods that explore sophisticated molecule and protein encodings tested in the present work, but that did not lead to improvements in prediction performance.

## Data Availability

Regarding the implementation, our code is available on GitHub at: https://github.com/bplaye/NNk_DTI. The datasets supporting the conclusions of this article are available in the data repository, http://members.cbio.mines-paristech.fr/~bplaye/NNk_DTI.zip. Also, we broadly used machine learning shallow algorithms and auxiliary functions (for instance for computing performance scores) implemented in the scikit-learn library [61], a viral and well-maintained free software machine learning library for Python. Also, we used the Tensorflow library [72] and the Keras library [73] to implement deep learning algorithms. To implement *NRLMF*, we used the PyDTI python package [23] available at: https://github.com/stephenliu0423/PyDTI. We used the implementation of LAkernel software [74] available at: http://members.cbio.mines-paristech.fr/~jvert/software/.

## Abbreviations

*FNN*: Feed-forward neural network
*MLP*: Multi-layer perceptron
*GCN*: Graph neural network
*CN*: Chemogenomic neural network
*CNN*: Convolutional neural network
*RNN*: Recurrent neural network
*LSTM*: Long-Short Term Memory (RNN)
*RLS*: Re-weighted Least Square
*NRLMF*: Neighbourhood Regularised Logistic Matrix Factorisation
*SVM*: Support vector machine

## References

[1] Drews J (2000). Drug discovery: a historical perspective. Science.

[2] Bleicher KH, Böhm H-J, Müller K, Alanine AI (2003). A guide to drug discovery: hit and lead generation: beyond high-throughput screening. Nat Rev Drug Disc.

[3] Brown RD, Martin YC (1997). The information content of 2d and 3d structural descriptors relevant to ligand-receptor binding. J Chem Inform Comput Sci.

[4] Azencott C-A (2010) Statistical machine learning and data mining for chemoinformatics and drug discovery. PhD thesis, University of California, Irvine

[5] Vert J-P, Jacob L (2008). Machine learning for in silico virtual screening and chemical genomics: new strategies. Comb Chem High Throughput Screen.

[6] Cortes-Ciriano I, van Westen GJ, Murrell DS, Lenselink EB, Bender A, Malliavin TE (2015). Applications of proteochemometrics-from species extrapolation to cell line sensitivity modelling. BMC Bioinform.

[7] van Westen GJ, Swier RF, Wegner JK, IJzerman AP, van Vlijmen HW, Bender A (2013). Benchmarking of protein descriptor sets in proteochemometric modeling (part 1): comparative study of 13 amino acid descriptor sets. J Cheminform.

[8] van Westen GJ, Swier RF, Cortes-Ciriano I, Wegner JK, Overington JP, IJzerman AP, van Vlijmen HW, Bender A (2013). Benchmarking of protein descriptor sets in proteochemometric modeling (part 2): modeling performance of 13 amino acid descriptor sets. J Cheminform.

[9] Yamanishi Y, Araki M, Gutteridge A, Honda W, Kanehisa M (2008). Prediction of drug–target interaction networks from the integration of chemical and genomic spaces. Bioinformatics.

[10] Jacob L, Vert J-P (2008). Protein–ligand interaction prediction: an improved chemogenomics approach. Bioinformatics.

[11] Bleakley K, Yamanishi Y (2009). Supervised prediction of drug–target interactions using bipartite local models. Bioinformatics.

[12] Yamanishi Y, Kotera M, Kanehisa M, Goto S (2010). Drug–target interaction prediction from chemical, genomic and pharmacological data in an integrated framework. Bioinformatics.

[13] Hizukuri Y, Sawada R, Yamanishi Y (2015). Predicting target proteins for drug candidate compounds based on drug-induced gene expression data in a chemical structure-independent manner. BMC Med Genom.

[14] Takarabe M, Kotera M, Nishimura Y, Goto S, Yamanishi Y (2012). Drug target prediction using adverse event report systems: a pharmacogenomic approach. Bioinformatics.

[15] Yamanishi Y (2013). Inferring chemogenomic features from drug–target interaction networks. Mol Inform.

[16] Yuan Q, Gao J, Wu D, Zhang S, Mamitsuka H, Zhu S (2016). Druge-rank: improving drug–target interaction prediction of new candidate drugs or targets by ensemble learning to rank. Bioinformatics.

[17] van Laarhoven T, Nabuurs SB, Marchiori E (2011). Gaussian interaction profile kernels for predicting drug–target interaction. Bioinformatics.

[18] van Laarhoven T, Marchiori E (2013). Predicting drug–target interactions for new drug compounds using a weighted nearest neighbor profile. PLoS ONE.

[19] Mei J-P, Kwoh C-K, Yang P, Li X-L, Zheng J (2013). Drug–target interaction prediction by learning from local information and neighbors. Bioinformatics.

[20] Xia Z, Wu L-Y, Zhou X, Wong ST (2010). Semi-supervised drug–protein interaction prediction from heterogeneous biological spaces. BMC Syst Biol.

[21] Zheng X, Ding H, Mamitsuka H, Zhu S (2013) Collaborative matrix factorization with multiple similarities for predicting drug–target interactions. In: Proceedings of the 19th ACM SIGKDD international conference on knowledge discovery and data mining. ACM, New York, pp 1025–1033

[22] Gönen M (2012). Predicting drug–target interactions from chemical and genomic kernels using bayesian matrix factorization. Bioinformatics.

[23] Liu Y, Wu M, Miao C, Zhao P, Li X-L (2016). Neighborhood regularized logistic matrix factorization for drug–target interaction prediction. PLoS Comput Biol.

[24] Jacob L, Hoffmann B, Stoven V, Vert J-P (2008). Virtual screening of gpcrs: an in silico chemogenomics approach. BMC Bioinform.

[25] Playe B, Azencott C-A, Stoven V (2017) Efficient multi-task chemogenomics for drug specificity prediction. bioRxiv, 193391 10.1371/journal.pone.0204999 PMC6171913 30286165

[26] Gonen Mehmet, Kaski Samuel (2014). Kernelized Bayesian Matrix Factorization. IEEE Transactions on Pattern Analysis and Machine Intelligence.

[27] Weininger D (1988). Smiles, a chemical language and information system. 1. Introduction to methodology and encoding rules. J Chem Inform Comput Sci.

[28] Kwon S, Yoon S (2017) Deepcci: End-to-end deep learning for chemical-chemical interaction prediction. In: Proceedings of the 8th ACM international conference on bioinformatics, computational biology, and health informatics, pp. 203–212. ACM

[29] Xu Z, Wang S, Zhu F, Huang J (2017) Seq2seq fingerprint: An unsupervised deep molecular embedding for drug discovery. In: Proceedings of the 8th ACM international conference on bioinformatics, computational biology, and health informatics, pp. 285–294. ACM

[30] Hamilton WL, Ying R, Leskovec J (2017) Representation learning on graphs: Methods and applications. arXiv preprint arXiv:1709.05584

[31] Hamilton W, Ying Z, Leskovec J (2017) Inductive representation learning on large graphs. In: Advances in neural information processing systems, pp. 1024–1034

[32] Duvenaud DK, Maclaurin D, Iparraguirre J, Bombarell R, Hirzel T, Aspuru-Guzik A, Adams RP (2015) Convolutional networks on graphs for learning molecular fingerprints. In: Advances in neural information processing systems, pp. 2224–2232

[33] Dai H, Dai B, Song L (2016) Discriminative embeddings of latent variable models for structured data. In: International conference on machine learning, pp. 2702–2711

[34] Lusci A, Pollastri G, Baldi P (2013). Deep architectures and deep learning in chemoinformatics: the prediction of aqueous solubility for drug-like molecules. J Chem Inform Model.

[35] Coley CW, Barzilay R, Green WH, Jaakkola TS, Jensen KF (2017). Convolutional embedding of attributed molecular graphs for physical property prediction. J Chem Inform Model.

[36] Li Y, Tarlow D, Brockschmidt M, Zemel R (2015) Gated graph sequence neural networks. arXiv preprint arXiv:1511.05493

[37] Altae-Tran H, Ramsundar B, Pappu AS, Pande V (2017). Low data drug discovery with one-shot learning. ACS Cent Sci.

[38] Gadiya S, Anand D, Sethi A (2018) Some new layer architectures for graph cnn. arXiv preprint arXiv:1811.00052

[39] Schlichtkrull M, Kipf TN, Bloem P, Van Den Berg R, Titov I, Welling M (2018). Modeling relational data with graph convolutional networks. European semantic web conference.

[40] Shang C, Liu Q, Chen K-S, Sun J, Lu J, Yi J, Bi J (2018) Edge attention-based multi-relational graph convolutional networks. arXiv preprint arXiv:1802.04944

[41] Kipf TN, Welling M (2016) Semi-supervised classification with graph convolutional networks. arXiv preprint arXiv:1609.02907

[42] Wang S, Weng S, Ma J, Tang Q (2015). Deepcnf-d: predicting protein order/disorder regions by weighted deep convolutional neural fields. Int J Mol Sci.

[43] Lyons J, Dehzangi A, Heffernan R, Sharma A, Paliwal K, Sattar A, Zhou Y, Yang Y (2014). Predicting backbone c$$\alpha $$ angles and dihedrals from protein sequences by stacked sparse auto-encoder deep neural network. J Comput Chem.

[44] Riis SK, Krogh A (1996). Improving prediction of protein secondary structure using structured neural networks and multiple sequence alignments. J Comput Biol.

[45] Sønderby SK, Winther O (2014) Protein secondary structure prediction with long short term memory networks. arXiv preprint arXiv:1412.7828

[46] Agathocleous M, Christodoulou G, Promponas V, Christodoulou C, Vassiliades V, Antoniou A (2010). Protein secondary structure prediction with bidirectional recurrent neural nets: can weight updating for each residue enhance performance?. IFIP international conference on artificial intelligence applications and innovations.

[47] Jurtz VI, Johansen AR, Nielsen M, Almagro Armenteros JJ, Nielsen H, Sønderby CK, Winther O, Sønderby SK (2017). An introduction to deep learning on biological sequence data: examples and solutions. Bioinformatics.

[48] Öztürk H, Özgür A, Ozkirimli E (2018). Deepdta: deep drug–target binding affinity prediction. Bioinformatics.

[49] He T, Heidemeyer M, Ban F, Cherkasov A, Ester M (2017). Simboost: a read-across approach for predicting drug–target binding affinities using gradient boosting machines. J Cheminform.

[50] Tsubaki M, Tomii K, Sese J (2018). Compound–protein interaction prediction with end-to-end learning of neural networks for graphs and sequences. Bioinformatics..

[51] Koutsoukas A, Monaghan KJ, Li X, Huan J (2017). Deep-learning: investigating deep neural networks hyper-parameters and comparison of performance to shallow methods for modeling bioactivity data. J Cheminform.

[52] Wishart DS, Knox C, Guo AC, Shrivastava S, Hassanali M, Stothard P, Chang Z, Woolsey J (2006). Drugbank: a comprehensive resource for in silico drug discovery and exploration. Nucl Acids Res.

[53] Bolton EE, Wang Y, Thiessen PA, Bryant SH (2008). Pubchem: integrated platform of small molecules and biological activities. Ann Rep Comput Chem.

[54] Gaulton A, Bellis LJ, Bento AP, Chambers J, Davies M, Hersey A, Light Y, McGlinchey S, Michalovich D, Al-Lazikani B (2012). Chembl: a large-scale bioactivity database for drug discovery. Nucleic Acids Res.

[55] Wu Z, Ramsundar B, Feinberg EN, Gomes J, Geniesse C, Pappu AS, Leswing K, Pande V (2018). Moleculenet: a benchmark for molecular machine learning. Chem Sci.

[56] Hanley JA, McNeil BJ (1982). The meaning and use of the area under a receiver operating characteristic (roc) curve. Radiology.

[57] Raghavan V, Bollmann P, Jung GS (1989). A critical investigation of recall and precision as measures of retrieval system performance. ACM Trans Inform Syst.

[58] Pahikkala T, Airola A, Pietilä S, Shakyawar S, Szwajda A, Tang J, Aittokallio T (2014) Toward more realistic drug–target interaction predictions. Briefings in bioinformatics, 010 10.1093/bib/bbu010 PMC4364066 24723570

[59] Saigo H, Vert J-P, Ueda N, Akutsu T (2004). Protein homology detection using string alignment kernels. Bioinformatics.

[60] Swamidass SJ, Chen J, Bruand J, Phung P, Ralaivola L, Baldi P (2005). Kernels for small molecules and the prediction of mutagenicity, toxicity and anti-cancer activity. Bioinformatics.

[61] Pedregosa F, Varoquaux G, Gramfort A, Michel V, Thirion B, Grisel O, Blondel M, Prettenhofer P, Weiss R, Dubourg V, Vanderplas J, Passos A, Cournapeau D, Brucher M, Perrot M, Duchesnay E (2011). Scikit-learn: machine learning in Python. J Mach Learn Res.

[62] van Westen GJ, Wegner JK, IJzerman AP, van Vlijmen HW, Bender A (2011). Proteochemometric modeling as a tool to design selective compounds and for extrapolating to novel targets. Med Chem Comm.

[63] Ong SA, Lin HH, Chen YZ, Li ZR, Cao Z (2007). Efficacy of different protein descriptors in predicting protein functional families. BMC Bioinform.

[64] Ramsundar B, Kearnes S, Riley P, Webster D, Konerding D, Pande V (2015) Massively multitask networks for drug discovery. arXiv preprint arXiv:1502.02072

[65] Ruder S (2017) An overview of multi-task learning in deep neural networks. arXiv preprint arXiv:1706.05098

[66] Paul A, Jha D, Liao W-k, Choudhary A, Agrawal A (2019) Transfer learning using ensemble neural nets for organic solar cell screening. arXiv preprint arXiv:1903.03178

[67] Gao KY, Fokoue A, Luo H, Iyengar A, Dey S, Zhang P (2018) Interpretable drug target prediction using deep neural representation. In: IJCAI, pp. 3371–3377

[68] Duan J, Sastry M, Dixon SL, Lowrie JF, Sherman W (2011). Analysis and comparison of 2d fingerprints: insights into database screening performance using eight fingerprint methods. J Cheminform.

[69] Bender A, Jenkins JL, Scheiber J, Sukuru SCK, Glick M, Davies JW (2009). How similar are similarity searching methods? a principal component analysis of molecular descriptor space. J Chem Inform Model.

[70] Riniker S, Landrum GA (2013). Open-source platform to benchmark fingerprints for ligand-based virtual screening. J Cheminform.

[71] Alberga D, Trisciuzzi D, Montaruli M, Leonetti F, Mangiatordi GF, Nicolotti O (2018). A new approach for drug target and bioactivity prediction: the multifingerprint similarity search algorithm (mussel). J Chem Inform Model..

[72] Abadi M, Barham P, Chen J, Chen Z, Davis A, Dean J, Devin M, Ghemawat S, Irving G, Isard M (2016). Tensorflow: a system for large-scale machine learning. OSDI.

[73] Chollet F et al (2015) Keras. https://keras.io

[74] Vert J-P (2008) The optimal assignment kernel is not positive definite. arXiv preprint arXiv:0801.4061

