# Supplementary material for: Evaluation of deep and shallow learning methods in chemogenomics for the prediction of drugs specificity
Source: J Cheminform. 2020 Feb 10;12:11. doi: 10.1186/s13321-020-0413-0 (PMC7011501; doi:10.1186/s13321-020-0413-0)
Supplement: Supplementary file 1 — Additional file 1. Tables presenting the ROCAUC and AUPR scores on the DBH and DBEC datasets for various positive:negative ratios. [file 13321_2020_413_MOESM1_ESM.pdf]

Additional file 1
Evaluation of deep and shallow learning methods in chemogenomics for the prediction of drugs specificity
Playe Benoit, Véronique Stoven

Introduction

In this Supplementary Materials, we report our investigations of modifications to the standard GNN formulation for molecules encoding, to the protein encoding neural network and to the combination of protein and molecule representations.

1 Exploring molecular graph encoding neural network

Among the modifications that have been proposed in the general field of graph representation learning, we considered those that appeared the most relevant in the context of chemogenomics, both in terms of intuition and expected performance improvements, as discussed below.

1.1 GAT

Among $AGGREGATE_{node}^{(l)}$ functions, the GAT function proposed by Velickovic et al. [1] seemed the most promising to us. Indeed, it resulted in significant performance improvement, and is intuitively relevant since it intends to prioritise relevant neighbours via a multi-head attention mechanism at each node update.

We used the original implementation provided by the authors of the GAT method. We optimised by varying the number of convolutional filters (in {10,20,50,100,1000}), the keeping probability of dropout (in {0,0.3,0.6}) and the number of attention heads (in {1,5,10}).

The GAT function did not lead to significant performance improvement for the four settings, as reported in Table 1. Although the GAT function led to substantial improvements in gold standard datasets of graph representation learning, it did not in chemogenomics. This may be due to the the fact that molecular graphs are small, and that all atoms of molecules are informative when learning an end-to-end molecule encoding.

1.2 Considering edge descriptors

We also assayed the "wbond" function as $AGGREGATE_{node}^{(l)}$ function that takes into account the bond attributes for the aggregation. The aim was to test whether bond attributes can bring representation power and flexibility. Here,

the "wbond" $AGGREGATE_{node}^{(l)}$ function refers to (as in Coley et al. [2]):

$$\mathbf{h}_i^{(l+1)} = \sigma(\mathbf{W}_0^{(l)} \cdot \mathbf{h}_i^{(l)} + \sum_{j \in \mathcal{N}(i)} \mathbf{W}_1^{(l)} \cdot [\mathbf{h}_j^{(l)}, \mathbf{x}_{ij}])$$

where σ is the sigmoid function, $\mathbf{h}_i^{(l+1)}$ is the representation of atom i at step $l+1$, $[\mathbf{h}_j^{(l)}, \mathbf{x}_{ij}]$ is the concatenation of the representation $\mathbf{h}_j^{(l)}$ of atom i at step l and the bond attribute vector $\mathbf{x}_{ij}$ between atoms i and j, and $\mathbf{W}_0^{(l)}, \mathbf{W}_1^{(l)}$ are learnable parameters.

We optimised by varying the number of convolutional filters (in {10,20,50,100,1000}) and the keeping probability of dropout (in {0,0.3,0.6}).

The "wbond" function did not lead to significant performance improvement for the four settings, as reported in Table 1. The standard GNN may already leverage bond information based on the nodes attributes and degrees (i.e., based on the topology of the graph).

Independently, we tested several options to replace the summation in the $AGGREGATE_{graph}^{(l)}$ function and standard $COMBINE_{graph}$ function of the minimal GNN, that builds the graph-level representation directly from node representations.

1.3 Max aggregation

When used as the $AGGREGATE_{graph}^{(l)}$ function, the sum function captures the distribution of node features in a single value. Alternatively, the max function is expected to capture representative elements in the node features' distributions, with no additional parameters to learn. This could be relevant for molecular bio-activity prediction, as bio-activity may rely on representative elements such as pharmacophores or functional groups. The chemogenomic neuron network, whose GNN's $AGGREGATE_{graph}^{(l)}$ function is set to the max function, is mentioned as "CN-MaxAgg" in Table 1. Note that even if the sum function can theoretically capture representative elements, in particular when the dimension of the feature vector is high, the max function enforces capturing representative elements, which may improve the performance.

However, it did not lead to substantial performance improvements for the four settings on the *DBEColi* dataset.

Intuitively, this means that, in the case of small chemical compounds, the simple sum function has the same representation power than the max for the $AGGREGATE_{graph}^{(l)}$ function. Therefore, in the case of small molecular graphs, the sum function can capture representative elements in the graph (as well as the max function), if these strategies are relevant for DTI prediction.

1.4 Concatenation combination

Regarding the $COMBINE_{graph}$ function, we tested concatenation of graph-level representations after each updated $\mathbf{m}^{(l)}$, used to build the final graph-level representation $\mathbf{m}$. We expect that it could help learning by skipping deleterious layers and by representing molecules via substructures of different sizes. The chemogenomic neuron network, whose GNN's $COMBINE_{graph}$ function is set to the concatenation function, is mentioned as "CN-ConcatCombine" in Table 1. Note that the simple sum function can, theoretically, adopt the same behaviour if the dimension of the feature vector is high enough, and if the network learns to store information in different locations in the feature vector at each update step.

This did not lead to substantial performance improvements for the four settings on the $DBEColi$ dataset.

Intuitively, this means that, in the case of small chemical compounds, the simple sum function has the same representation power than the concatenation for the $COMBINE^{graph}$ function. Therefore, in the case of small molecular graphs, the sum function can learn to store information of subgraphs of different sizes (as well as the concatenation function), if these strategies are relevant for DTI prediction.

1.5 Hierarchical pooling combination

A promising approach to define a suitable $AGGREGATE^{(l)}_{graph}$ function is based on differential hierarchical pooling, which has been proposed simultaneously by several groups [3, 4, 5]. It allows for the graph convolutional architecture to iteratively operate on coarser representations of a graph. To be precise, such procedure actually replaces both the $AGGREGATE^{(l)}_{graph}$ and $COMBINE_{graph}$ functions, since it results directly in a final graph-level representation. This idea was to design an analogue of the pooling operation for images, which was shown to be of crucial importance for convolutional neuron network success in the field of image processing. Indeed, one of the possible limitations of current GNNs is that they are inherently flat, since they only propagate information across the edges of the graph, without reducing the size of the graph. However, this could be not particularly suitable for molecular graphs. Indeed, they are small enough so that iterative convolutional node updates, followed by a simple summation of graph nodes to build a graph-level embedding, could equivalently encode and hierarchically agglomerate graph substructures of different sizes. Therefore, we discuss and evaluate differential hierarchical pooling in more details later in this work.

The chemogenomic neuron network whose GNN is based on hierarchical pooling is mentioned as "CN-pool" in Table 1. Again, note that a simple sum over node feature vectors can mimic the same behaviour, but the hierarchical pooling procedure is expected to enforce learning the graph-level representation in a hierarchical fashion and, hence, to improve encoding.

This did not lead to substantial performance improvements for the four settings on the *DBEColi* dataset.

It seems that standard GNN can also leverage information of substructures of different sizes in a hierarchical manner. Indeed, the iteration of the nodes update can already provide neighbouring information to each node in a hierarchical manner.

2 Exploring protein sequence encoding neural network

Since a priori relevant modifications of the GNN architecture encoding molecular graphs did not significantly improve the prediction performance of the resulting chemogenomic network, the performance bottleneck could be related to the protein sequence encoder rather than the molecular graph encoder.

Therefore, we replaced the stacked CNN block that encodes the proteins in the standard chemogenomic network by a biLSTM layer on top of a convolution layer. Indeed, the bi-LSTM can locally integrate the detected patterns by the convolutional layer to provide local contextual information, both forward and backwards, whereas stacked convolutional layers process protein sequences by detecting local patterns, patterns of patterns and so on.

We varied the dimension of the amino acids learnt representations ({10, 50, 100, 500}) and the amount of dropout ({0., 0.1, 0.3, 0.6}), to search for the best performance.

However, the best performance reached by this architecture was identical that obtained while using stacked convolutional layers of the standard chemogenomic network, as shown in Table 1 at the row named "CN-biLSTM".

3 Exploring protein and molecule representations combination

We now propose to investigate the use of an attention mechanism in the CNN protein sequence encoder, which modifies the simple concatenation used as *Comb* operation in the standard chemogenomic network.

We first implemented the attention mechanism proposed by Tsubaki et al [6] and displayed in eq. **??**, which resulted in a significant decrease of performance for the four S_1, S_2, S_3, and S_4 settings. This might be due to the fact that this attention mechanism is highly non-convex, since it relies on the dot product of the two learnt representations, resulting in a rugged optimisation landscape.

Therefore, instead of using an attention mechanism calculated only for the amino acids representations, we turned to an attention mechanism that computes the pairwise representation $\mathbf{h}_{pair}$ based on the concatenation $[\mathbf{h}_{prot}, \mathbf{h}_{mol}]$ of the protein $\mathbf{h}_{prot}$ and molecule $\mathbf{h}_{mol}$ learnt representations, following:

$$\mathbf{h}_{pair} = \sigma(\mathbf{W}_{att} \cdot [\mathbf{h}_{prot}, \mathbf{h}_{mol}] + \mathbf{b}_{att}) \odot [\mathbf{h}_{prot}, \mathbf{h}_{mol}]$$

In the previous equation, σ refers to the sigmoid function and $\odot$ to the element-wise multiplication. This self-attention mechanism differs from that of Tsubaki et al. in two ways. First, it selects learnt abstract features at the protein and molecule level, whereas the precedent attention mechanism learnt attention weights on abstract features at the amino acid level. Second, this mechanism operates on the concatenation of the protein and molecule abstract features, and jointly selects the most important protein and molecule features with respect to the prediction task. This appears rather intuitive since the protein-ligand interaction mechanism relies on both partners. However, in the present case, the selected features are abstract, and therefore, not easily interpretable.

As reported in Table 1 (at the row named "CN-pairwiseAtt"), this did not result in performance improvement, meaning that such soft attention mechanism does not provide better representation power. It is most likely that the self-attention mask "$\sigma(\mathbf{W}_{att} \cdot [\mathbf{h}_{prot}, \mathbf{h}_{mol}] + \mathbf{b}_{att})$" produces a uniform attention weight distribution in most cases.

		raw (S_1)	orphan proteins (S_2)	orphan molecules (S_3)	double orphan (S_4)
	standard chemogenomic neuron network (CN)	39.57 ± 4.17	26.74 ± 2.49	43.74 ± 2.35	24.63 ± 1.89
change in molecular graph encoding	CN-GAT	39.97 ± 4.64	25.84 ± 3.58	43.53 ± 2.35	26.28 ± 3.72
	CN-wbond	35.65 ± 1.47	23.27 ± 1.97	34.38 ± 3.87	25.01 ± 4.14
	CNN-MaxAgg	38.33 ± 2.85	26.24 ± 2.29	46.06 ± 2.28	21.57 ± 2.41
	CN-ConcatCombine	43.77 ± 3.59	26.04 ± 2.29	44.31 ± 2.45	24.97 ± 3.50
	CN-pool	40.54 ± 6.82	18.71 ± 0.53	36.63 ± 7.95	19.24 ± 2.60
change in protein encoding	CN-biLSTM	40.65 ± 1.88	28.69 ± 1.55	41.55 ± 3.08	22.79 ± 2.72
change in protein and molecular representations combinations	CN-pairwiseAtt	35.76 ± 2.15	23.84 ± 5.15	41.96 ± 2.92	22.69 ± 7.21

Table 1: AUPR score of various modifications of the chemogenomic neuron network on a single train/validation/test split of the *DBEColi* dataset. Standard deviations are obtained after repeating 5 times the evaluation procedure.

References

[1] Petar Velickovic, Guillem Cucurull, Arantxa Casanova, Adriana Romero, Pietro Lio, and Yoshua Bengio. Graph attention networks. *arXiv preprint arXiv:1710.10903*, 2017.

[2] Connor W Coley, Regina Barzilay, William H Green, Tommi S Jaakkola, and Klavs F Jensen. Convolutional embedding of attributed molecular graphs for physical property prediction. *Journal of chemical information and modeling*, 57(8):1757–1772, 2017.

[3] Rex Ying, Jiaxuan You, Christopher Morris, Xiang Ren, William L Hamilton, and Jure Leskovec. Hierarchical graph representation learning withdifferentiable pooling. *arXiv preprint arXiv:1806.08804*, 2018.

[4] Angelo Porrello, Davide Abati, Simone Calderara, and Rita Cucchiara. Classifying signals on irregular domains via convolutional cluster pooling. *arXiv preprint arXiv:1902.04850*, 2019.

[5] Shrey Gadiya, Deepak Anand, and Amit Sethi. Some new layer architectures for graph cnn. *arXiv preprint arXiv:1811.00052*, 2018.

[6] Masashi Tsubaki, Kentaro Tomii, and Jun Sese. Compound-protein interaction prediction with end-to-end learning of neural networks for graphs and sequences. *Bioinformatics*, 2018.
