# Supplementary material for: Evaluation of deep and shallow learning methods in chemogenomics for the prediction of drugs specificity
Source: J Cheminform. 2020 Feb 10;12:11. doi: 10.1186/s13321-020-0413-0 (PMC7011501; doi:10.1186/s13321-020-0413-0)
Supplement: Supplementary file 2 — Additional file 2. Principles and results of several promising methods that explore sophisticated molecule and protein encodings tested in the present work, but that did not lead to improvements in prediction performance. [file 13321_2020_413_MOESM2_ESM.pdf]

Additional File 2

	raw (S_1)			orphan proteins (S_2)			orphan molecules (S_3)			double orphan (S_4)		
	1:1	1:2	1:5	1:1	1:2	1:5	1:1	1:2	1:5	1:1	1:2	1:5
SVM	**75.14 ± 1.7**	74.95 ± 2.04	74.99 ± 1.76	54.97 ± 4.33	56.5 ± 4.29	56.67 ± 4.44	74.03 ± 3.8	73.89 ± 3.78	74.14 ± 3.72	53.84 ± 8.86	54.46 ± 8.43	53.06 ± 9.63
NRLMF	**78.8 ± 3.68**	**78.88 ± 2.73**	**78.41 ± 2.69**	**65.05 ± 7.1**	**64.96 ± 6.32**	**65.4 ± 4.9**	**77.19 ± 1.65**	**77.21 ± 2.06**	**77.38 ± 1.81**	**65.2 ± 7.26**	**64.59 ± 6.26**	**63.61 ± 6.51**
FNN	**75.92 ± 1.17**	74.63 ± 1.04	74.88 ± 1.52	**58.25 ± 5.34**	55.12 ± 4.67	54.89 ± 4.98	74.67 ± 2.38	**75.52 ± 2.4**	73.96 ± 3.15	56.03 ± 9.22	55.91 ± 8.07	54.68 ± 8.12
chemogenomic neuron network	**77.1 ± 1.83**	**76.5 ± 0.89**	**76.74 ± 0.85**	**61.9 ± 4.41**	**59.64 ± 8.55**	**64.25 ± 4.0**	73.49 ± 3.99	74.04 ± 4.91	74.91 ± 3.98	**59.23 ± 9.5**	**58.4 ± 8.74**	**58.62 ± 9.1**

Table S1 : ROCAUC scores on the *DBEC* dataset in four settings, for a test sample positive:negative ratios in {1:1, 1:2, 1:5}

	raw (S_1)			orphan proteins (S_2)			orphan molecules (S_3)			double orphan (S_4)		
	1:1	1:2	1:5	1:1	1:2	1:5	1:1	1:2	1:5	1:1	1:2	1:5
SVM	**81.75 ± 1.75**	**73.25 ± 2.4**	**61.55 ± 3.11**	59.62 ± 5.55	**45.89 ± 6.73**	**28.95 ± 7.66**	**80.79 ± 2.37**	**72.02 ± 3.06**	**60.96 ± 3.73**	58.52 ± 9.21	43.37 ± 9.15	24.4 ± 8.8
NRLMF	**82.56 ± 2.82**	**73.89 ± 2.98**	**59.89 ± 4.35**	**67.56 ± 7.04**	**53.18 ± 8.12**	**35.62 ± 8.07**	**81.49 ± 1.48**	**72.35 ± 2.2**	**60.06 ± 2.73**	**67.93 ± 8.63**	**52.97 ± 8.7**	**34.5 ± 9.75**
FNN	**79.82 ± 0.98**	68.07 ± 2.35	51.55 ± 2.55	**61.92 ± 5.11**	42.24 ± 5.27	24.26 ± 3.91	78.57 ± 2.22	68.14 ± 3.43	49.34 ± 3.11	**60.04 ± 8.83**	43.55 ± 9.84	22.97 ± 6.59
chemogenomic neuron network	79.04 ± 2.24	66.5 ± 2.51	49.08 ± 4.31	**60.79 ± 6.24**	**45.11 ± 8.65**	**28.38 ± 5.81**	76.23 ± 2.23	64.61 ± 4.78	46.14 ± 4.92	**61.97 ± 8.72**	**45.81 ± 8.5**	**27.0 ± 6.96**

Table S2 : AUPR scores on the *DBEC* dataset in four settings, for a test sample positive:negative ratios in {1:1, 1:2, 1:5}

	raw (S_1)			orphan proteins (S_2)			orphan molecules (S_3)			double orphan (S_4)		
	1:1	1:2	1:5	1:1	1:2	1:5	1:1	1:2	1:5	1:1	1:2	1:5
SVM	74.6 ± 0.57	74.6 ± 0.55	74.62 ± 0.54	50.26 ± 3.05	49.88 ± 3.06	49.88 ± 2.95	75.16 ± 1.51	75.28 ± 1.76	75.14 ± 1.69	49.79 ± 1.61	50.25 ± 1.84	50.5 ± 1.68
NRLMF	82.98 ± 0.55	83.06 ± 0.65	83.12 ± 0.63	65.18 ± 1.36	65.18 ± 1.28	65.23 ± 1.2	59.49 ± 0.96	59.56 ± 0.81	59.6 ± 0.93	50.37 ± 1.48	50.29 ± 1.55	50.47 ± 1.35
FNN	**90.39 ± 0.42**	**90.65 ± 0.46**	**90.45 ± 0.52**	74.74 ± 0.4	74.08 ± 2.29	74.25 ± 1.96	83.06 ± 1.48	**83.52 ± 1.52**	82.9 ± 1.8	**69.12 ± 2.3**	**69.17 ± 1.99**	68.79 ± 1.69
chemogenomic neuron network	88.67 ± 1.59	88.21 ± 1.47	88.21 ± 2.23	**81.5 ± 1.47**	**80.83 ± 2.7**	**80.28 ± 1.4**	**85.76 ± 1.5**	**85.4 ± 2.02**	**85.76 ± 2.23**	**73.34 ± 5.89**	**71.21 ± 5.53**	**73.68 ± 4.87**

Table S3 : ROCAUC scores on the *DBH* dataset in four settings, for a test sample positive:negative ratios in {1:1, 1:2, 1:5}

	raw (S_1)			orphan proteins (S_2)			orphan molecules (S_3)			double orphan (S_4)		
	1:1	1:2	1:5	1:1	1:2	1:5	1:1	1:2	1:5	1:1	1:2	1:5
SVM	78.34 ± 0.44	66.85 ± 0.68	48.99 ± 0.5	50.35 ± 2.64	33.46 ± 2.33	16.82 ± 1.54	78.2 ± 1.77	66.86 ± 2.12	48.66 ± 2.74	49.84 ± 1.7	33.26 ± 1.64	16.81 ± 1.02
NRLMF	86.14 ± 0.45	78.73 ± 0.75	66.93 ± 1.06	68.19 ± 1.55	53.68 ± 1.72	34.75 ± 1.65	61.88 ± 0.99	45.93 ± 0.72	26.42 ± 0.52	50.45 ± 1.31	33.71 ± 1.25	16.99 ± 0.8
FNN	**91.45 ± 0.45**	**86.5 ± 0.44**	**75.91 ± 0.49**	78.16 ± 0.44	66.02 ± 1.86	**49.13 ± 2.58**	**85.69 ± 1.05**	**78.15 ± 1.18**	**64.06 ± 1.6**	**71.46 ± 2.02**	**57.93 ± 2.7**	**38.61 ± 2.29**
chemogenomic neuron network	88.74 ± 1.68	80.29 ± 2.65	65.46 ± 4.53	**81.82 ± 1.9**	**70.39 ± 3.66**	**47.68 ± 4.38**	**86.0 ± 1.85**	**77.02 ± 3.41**	62.22 ± 4.66	**72.26 ± 6.42**	**55.98 ± 7.67**	**38.48 ± 7.5**

Table S4 : AUPR scores on the *DBH* dataset in four settings, for a test sample positive:negative ratios in {1:1, 1:2, 1:5}
